# Bacteroides ovatus-derived N-methylserotonin inhibit colorectal cancer via the HTR1D-mediated cAMP-PKA-NF-κB signaling axis

**DOI:** 10.3389/fimmu.2025.1696701

**Published:** 2025-11-24

**Authors:** Jikai He, Jiaqi Jia, Wenhao Qu, Shuyu Zhang, Keyu Fan, Runze Lin, Wei Zhao, Yan Niu, Yanqiang Huang, Lizhou Jia

**Affiliations:** 1 Graduate School of Youjiang Medical University for Nationalities, Baise, China; 2 Clinical Laboratory, Zhijin County People's Hospital, Bijie, China; 3 School of Arts and Sciences, University of Rochester, Rochester, NY, United States; 4 Department of Anesthesiology, Peking University People's Hospital, Beijing, China; 5 School of Radiation Medicine and Protection, Medical College of Soochow University, Suzhou, China; 6 Inner Mongolia Medical University, Hohhot, Inner Mongolia, China; 7 Central Laboratory of Bayannur Hospital, Bayannur, Inner Mongolia, China

**Keywords:** N-methylserotonin, bacteroides ovatus, colorectal cancer, gut microbiota, metabolites

## Abstract

**Objective:**

To analyze differences in gut microbiota composition, metabolites, and metabolic pathways between healthy individuals and colorectal cancer (CRC) patients, and to investigate the inhibitory effects of N-methylserotonin (NMS) produced by *Bacteroides ovatus* (B.o) from orange fiber on CRC progression and its underlying mechanisms.

**Methods:**

(1) Fecal samples from CRC patients (n=26) and healthy controls (n=20) were collected for metagenomic sequencing and untargeted metabolomics analysis; (2) The ability of B.o to produce NMS from orange fiber was validated *in vitro*; (3) A CRC mouse model was established using azoxymethane (AOM)/dextran sulfate sodium (DSS) induction, followed by evaluation of body weight, rectal bleeding, colorectal length, tumor number, and intestinal barrier function; (4) Network pharmacology, molecular docking, and western blot analysis were combined to verify the mechanism of action; (5) 16S rRNA sequencing was performed to analyze gut microbiota changes.

**Results:**

(1) CRC patients showed significantly increased metabolic pathways including glycolysis, methane metabolism, beneficial amino acid degradation, and linoleic acid degradation, along with significantly decreased B.o abundance and NMS levels, which were positively correlated; (2) NMS significantly inhibited CRC cell proliferation, migration, and invasion, while promoting apoptosis; (3) Combined treatment with B.o and orange fiber or NMS alone reduced tumorigenesis and improved intestinal barrier function; (4) Mechanistic studies revealed that these effects could be mediated through downregulation of 5-hydroxytryptamine receptor 1D (HTR1D) expression and inhibition of the cAMP/PKA/IκBα/NF-κB pathway; (5) The treatments optimized gut microbiota structure and metabolite composition.

**Conclusion:**

B.o and its metabolite NMS possibly inhibit CRC progression by modulating the HTR1D-mediated cAMP/PKA/NF-κB signaling pathway, while improving gut microbiota structure, providing a novel therapeutic target for CRC prevention and treatment.

## 1 Introduction

Colorectal cancer (CRC) is one of the most common malignant tumors worldwide, with an incidence of 6.0% and a mortality rate of 5.8% (1). Recent studies have demonstrated that the gut microbiota and its metabolites play a crucial role in the initiation, progression, early diagnosis, treatment, and prognosis of CRC (2–5). These microbiota and metabolites not only contribute to maintaining intestinal homeostasis and protecting mucosal integrity but also enhance immune function, regulate substance metabolism, and play crucial roles in the prevention and treatment of various diseases (6–8). Therefore, identifying novel beneficial metabolites and probiotics is of significant importance for preventing and treating CRC.

*Bacteroides ovatus* (B.o) has been identified as the most effective gut microbiota species for decomposing orange fiber to release NMS. This process, which involves breaking down pectin polysaccharides in orange fiber to produce NMS, is dependent on specific enzymes generated by B.o (9). Research by Professor Jeffrey’s team has shown that NMS, an endogenous substance found in plants and mammals, is produced by *Bacteroides_ovatus* during orange fiber metabolism. Oral administration of N-methylserotonin (NMS) to germ-free mice reduces obesity, increases hepatic glycogen synthesis, shortens intestinal transit time, and alters the expression of circadian genes in the liver and colon (9). Recent studies have demonstrated that B.o can metabolize dietary polysaccharides into monosaccharides. Consequently, B.o colonization has been shown to inhibit colonic mucus degradation, reduce disease severity in mouse graft-versus-host disease models, and improve survival rates (10). Furthermore, B.o can suppress taurocholic acid-mediated germination of *Clostridioides difficile*, showing potential for treating recurrent *C. difficile* infections (11). Additionally, B.o has been found to elevate intestinal hyodeoxycholic acid levels by increasing the abundance of *Flavonifractor* spp, thereby preventing renal fibrosis (12). *In vitro* experiments revealed that B.o synthesizes the neuroactive compound glutamine and γ-aminobutyric acid (GABA) through tryptophan and glutamate metabolism (13). In mouse lung cancer models, oral gavage of B.o significantly enhanced the efficacy of erlotinib, while inducing chemokine ligand 9 and interferon-gamma production (14). Serotonin (5-hydroxytryptamine) is a critical neurotransmitter regulating mood and sleep (15) NMS, a derivative of serotonin, is structurally similar to serotonin and is commonly used as an internal standard in serotonin assays (16). It also acts as an agonist for 5-hydroxytryptamine receptor 2A, increasing glucose uptake by skeletal muscle cells in experimental rats (17). Although these findings suggest potential antitumor effects of both B.o and NMS, there are currently no reported studies investigating B.o-derived NMS for cancer treatment.

## 2 Materials and methods

### 2.1 Main research materials

The study materials primarily included human normal colon epithelial cells (NCM460) (Fenghui Biological Cell Center), human CRC cell lines (HCT116, HT29) (Servicebio), and mouse CRC cell line CT26.wt (Servicebio), B.o (ATCC8483), NMS (Santa Cruz), orange fiber (Fiber Star, Citri-Fi100FG),azoxymethane (Aladdin), sodium dextran sulfate (Shanghai Yuanye), NF-κb antibodies (Abmart, T55034), IκBα antibodies (Abmart, T55026), Occludin antibodies (Abmart, TD7504), p53 antibodies (Abmart, T40060), cAMP antibodies (Abcam, ab76238), PKA antibodies (Affinit, AF7746), HTR1D antibodies (Affinit, DF2706), Ki-67 antibodies (MXB biotechnologies, RAM-0731), as well as cell cycle and apoptosis detection kits (Beyotime).

### 2.2 Research object

This study enrolled a total of 26 CRC patients diagnosed between July 2021 and December 2022 at the People’s Hospital of Bayannur, Inner Mongolia, China. Additionally, 20 healthy individuals (H) were included as controls, and listed in **Supplementary Table S1**. Inclusion criteria were: (1) initial diagnosis of CRC without prior radiotherapy, chemotherapy, or surgical treatment; (2) exclusion of intestinal-related diseases such as concurrent intestinal infections and inflammatory bowel disease, as well as metabolic disease; (3) all patients provided informed consent. The study was approved by the Ethics Committee of Bayannur Municipal Hospital (2020GG0273). Exclusion criteria were:(1) pregnancy, lactation, or menstruation females; (2) history of intestinal pathogen infections; (3) antibiotic/probiotic/prebiotic use within one month prior to specimen collection.

### 2.3 Metagenomic sequencing and annotation

Total DNA was extracted from fecal samples. The quality of extracted DNA was detected and quantified. The Macrogenomic sequencing, sequencing library construction, and high-throughput sequencing were performed at Hangzhou Lianchuan Biotechnology Co., Ltd. The raw sequencing data were processed by removing adapters and trimming low-quality sequences. In the circumstances of known host sequences, host sequences were removed. Individual samples were assembled, and the assembled contigs were subjected to CDS prediction. Clustering and redundancy removal were performed using CD-HIT based on the prediction results, then mapped the reads from each sample to the CDS sequence library for calculating the TPM abundance. Low-abundance expressions were filtered to obtain Unigenes. The Unigenes were compared to the NR mate library to obtain species annotation information. Furthermore, the Unigenes were compared and functionally annotated against the Gene ontology (GO), Kyoto encyclopedia of genes and genomes (KEGG) databases.

### 2.4 Fecal metabolite extraction and liquid chromatography tandem mass spectrometry analysis

Non targeted metabolomics was conducted at Hangzhou Lianchuan Biotechnology Co., Ltd.A high-resolution tandem mass spectrometer Q-Exactive was used to detect metabolites eluted form the column. The Q-Exactive was operated in both positive and negative ion modes. Precursor spectra were collected at 70,000resolution to hit an AGC target of 3e6. The maximum inject time was set to 100ms. A top 3 configuration to acquire data was set in DDA mode. Fragment spectra were collected at 17,500resolution to hit an AGC target of 1e5 with a maximum inject time of 80ms. In order to evaluate the stability of the LC-MS during the whole acquisition, a quality control sample was acquired after every 10 samples.

Finally, the raw data from the mass spectrometer were converted into readable data in mzXML format. The extracted substances were annotated with adduct ions, and then subjected to the first-level identification. Identification was performed using the first-level information from the mass spectrometry data, and the second-level information was matched with an in-house standard compound database. Annotations were provided for the candidate identification substances, elucidating the physical and chemical properties as well as biological functions of the metabolites. Quantification and screening of differential metabolites were conducted.

### 2.5 16S rRNA sequencing of the mouse feces

DNA extraction was performed on the fecal samples, and the integrity of the extracted DNA was verified. PCR amplification was carried out on qualified samples, followed by recovery and quantification of the amplification products. Subsequently, libraries were constructed and sequenced using high-throughput platforms. Raw sequencing data underwent filtering, assembly, and clustering to obtain Operational Taxonomic Units (OTUs), which were then annotated by comparison with databases.

### 2.6 Bioinformatics analysis

Alpha diversity analysis was performed using the Wilcoxon test. Principal coordinate analysis (PCoA) based on Bray-Curtis distance was performed to analyze the differences in microbiota and metabolites between healthy individuals and patients with CRC. Permutational multivariate analysis of variance (PERMANOVA) was used to analyze the explanatory degree of different groups to sample differences. Among different groups, differences in metabolite were analyzed using the Wilcoxon test, combined with Variable Important for the Projection (VIP) values derived from multivariate statistical analysis. Wilcoxon test was used to analyze the differences in gut microbiota between two groups, and Kruskal-Wallis rank-sum test was used to analyze the differences in gut microbiota among multiple groups. For LEfSe analysis, the Kruskal-Wallis rank-sum test was used to screen for differential species (The P value undergoes multiple comparisons to obtain the FDR value), and then the Wilcoxon rank-sum test was employed to determine whether all subspecies of significantly different species converged to the same classification level, use linear discriminant analysis (LDA) to obtain the final differentially expressed species. We utilized random forest analysis to do 10-fold cross-validation, and then constructed models for differentiating the H group from the CRC groups using differential microbiota and differential metabolites separately. All correlation analyses were conducted using Spearman’s correlation coefficient.

### 2.7 *In vitro* and *in vivo* NMS production from orange fiber by B.o

Bacterial suspensions were adjusted to 1.0×10^6^ CFU/mL and equally divided into two groups: (1) B.o group, and (2) B.o + orange fiber group (50 mg/mL, UV-sterilized CitriFi 100, FiberStar), with orange fiber alone as the control. Mixtures were incubated anaerobically without agitation for 72 hours, followed by NMS quantification (200 μL aliquots).

For *in vivo* studies, three 9-week-old specific-pathogen-free (SPF) C57BL/6J mice per group received: (1) standard chow, (2) B.o (ATCC 8483, 10^9^ CFU/kg/day), (3) orange fiber (CitriFi 100, 10 mg/mL in drinking water), or (4) B.o + orange fiber combination. After 7 days, fecal samples were collected in sterile Eppendorf tubes, wrapped in foil, flash-frozen in liquid nitrogen, and stored at -80 °C until NMS analysis.

### 2.8 CCK-8 and clone formation experiment

CCK-8 Assay: A total of 1,000 cancer cells were seeded into each well of a 96-well plate. After 24 hours of incubation, the medium was discarded, and different concentrations of NMS-containing media (100 μL per well) were added. After 48 hours, 10 μL of CCK-8 reagent was added to each well, and the plates were incubated for 2 hours. The absorbance at 450 nm was measured to assess cell viability.

Colony Formation Assay: The cells of each group were planted in a 6-well plate with 2000 cells/well. After 24 hours, the medium was replaced with medium containing 1 mmol concentration of the NMS. Two weeks later, the cells were fixed with formaldehyde and stained with 1% crystal violet. The colonies were then washed three times with PBS and photographed.

### 2.9 Migration and invasion experiments

Migration Assay: The cells of each group were planted in a 6-well plate with 80000 cells/well. After 24 hours of incubation, a scratch was created across the wells with a pipette tip, and the medium was discarded. The cells were then cultured in 2 mL of medium containing 1 mmol concentration of the NMS. At 0 h, 12 h, 24 h, 36 h, and 72 h, the cell migration was observed using an inverted fluorescent microscope and photographed.

Invasion Assay: Cells were suspended in serum-free medium at a concentration of 1×10^6^ cells/ml. The cells were then added to the upper chamber with a 1:8 dilution of Matrigel (100 μL per well). The lower chamber was filled with 500 μL of medium containing 20% serum and 1 mmol concentration of the NMS. After 48 hours of incubation, the cells were fixed with formaldehyde and stained with 0.1% crystal violet. The chamber was then washed three times with PBS and photographed.

### 2.10 Cell cycle and apoptosis experiments

First, all cells were collected and washed with EDTA. After resuspending in PBS, they were centrifuged at 1000 rpm for 5 minutes, and the supernatant was discarded. Subsequently, cell cycle and apoptosis rates were assessed following the instructions provided in the Cell Cycle and Apoptosis Detection Kit (Beyotime).

### 2.11 Network pharmacology analysis

NMS chemical structures and SMILES identifiers were retrieved from PubChem (https://pubchem.ncbi.nlm.nih.gov/). Potential targets were predicted using SwissTargetPrediction (http://www.swisstargetprediction.ch/). CRC-related genes were identified from GeneCards (www.genecards.org/) using “Colorectal cancer” as the keyword. Intersecting targets were analyzed via DAVID Bioinformatics Resources (https://davidbioinformatics.nih.gov/) for Gene Ontology (GO) and KEGG pathway enrichment.

### 2.12 Animal model

An inflammation-associated CRC model was established in SPF C57BL/6 mice (n=8/group) using AOM/DSS, with mice randomly divided into three groups: (1) Control: normal drinking water. (2) AOM/DSS: intraperitoneal AOM (10 mg/kg) at day 0, followed by 2% DSS for 7 days (week 2), then normal water for 2 weeks (3 cycles total). (3) Treatment groups: same protocol with interventions: Orange fiber (10 mg/mL), B.o (10^9^ CFU/kg/day), B.o + orange fiber, NMS (50 mg/kg/day) (9).

Body weight, survival, and hematochezia were monitored. At week 12, mice were euthanized for evaluation of the tumor burden, colorectal length, and hepatosplenic morphology.

### 2.13 Hematoxylin-eosin staining and immunohistochemistry

All tissue samples were fixed with 4% formalin and embedded in paraffin. Sections are placed in xylene to remove the paraffin wax. Tissue sections are stained with hematoxylin, which binds to acidic components in the tissue, such as nucleic acids, giving them a blue-purple color. After rinsing, the sections are stained with eosin, which stains basic components such as cytoplasm and extracellular matrix, giving them a pink-orange color. Sections are dehydrated with alcohol, cleared with xylene.

Sections of the tissue blocks were placed on glass slides and deparaffinized in gradient ethanol. Antigen repair was performed by heating in citrate buffer (0.01M, pH 6.0), followed by blocking of endogenous peroxidase activity using 3% hydrogen peroxide. The sections were then incubated by using primary antibodies, followed by secondary antibody incubation. Next, diaminobenzidine staining and hematoxylin counterstaining were performed, followed by hydrochloric acid ethanol differentiation. Finally, gradient ethanol and xylene were used for decolorization, and neutral resin was used for sealing. After completion, immunohistochemical evaluation needed to be performed. Investigators were blinded during the histological and immunohistochemical assessments.

### 2.14 Western blot

Extract total proteins from mouse colorectal tissue homogenate using radio immunoprecipitation Assay Lysis buffer (RIPA) buffer. Protein samples were mixed with loading buffer. The same amounts of proteins were separated by sodium dodecyl sulfate-polyacrylamide gel electrophoresis (SDS-PAGE) and transferred to a polyvinylidene fluoride (PVDF) membrane. Incubate the membrane with primary antibodies at 4°C overnight. After three rinses with PBST buffer for 15min each time, incubate the membrane with horseradish-peroxidase-labeled secondary antibody at room temperature for 2h. Then, the membrane was rinsed with PBST and developed.

### 2.15 Statistical analysis

Data were analyzed using GraphPad Prism 8. Normally distributed continuous variables were expressed as the mean ± standard deviation (SD; paired *t*-test for two groups, one-way ANOVA for multiple groups). Non-normal data were presented as the median (interquartile range, IQR) (Wilcoxon or Kruskal–Wallis rank sum test). Comparisons of baseline characteristics in **Supplementary Table S1** were performed using the independent t-test for continuous data and the Chi-square test (or Fisher’s exact test) for categorical data. Two-tailed tests with P<0.05 were considered significant. Symbols: *vs CRC group; #*vs* control; +*vs* B.o+fiber group. *P<0.05, **P<0.01, ***P<0.001, ****P<0.0001; #/+ follow same convention.

## 3 Result

### 3.1 Analysis of gut microbiota diversity and differential composition

To investigate the differences in the community structure and abundance of gut microbiota among the CRC and H groups, we performed Alpha diversity analysis. The results showed of Chao1 and Shannon analysis no statistically significant differences in gut microbiota diversity among the two groups (P > 0.05), indicating that there were no significant differences in gut microbiota diversity among the two groups (**Supplementary Figure S1A, B**). To further investigate the overall differences in gut microbiota composition among the different groups, we performed Beta diversity analysis to reflect the differences in species distribution and composition between groups. The results showed significant differences in species distribution and composition between the CRC, and H groups (CRC:H PERMANOVA, P = 0.001) (**Supplementary Figure S1C**). We further performed differential analysis and selected differential microbiota at the genus level in the gut microbiota. The results showed that compared to the H group, the CRC group had increased abundance of *Escherichia*, *Fusobacterium*, *Klebsiella*, *Lachnoclostridium*, *Shigella*. The abundance of *Lactococcus*, *Lachnospira*, *Eubacterium*, *Ruminococcus*, and other bacterial groups increased in H group (P < 0.05) (**Supplementary Figure S2A**, **Supplementary Table S2**). Furthermore, using LEfSe analysis to identify species with significant differences in study groups, we found that compared to the H group, the significant enrichment microbiota in the CRC group were *g:Klebsiella*, *s:Klebsiella_pneumoniae*, *g:Escherichia*, *s:Escherichia_colia*, *g:Fusobacterium*, *g:Porphyromonas*, *s:Porphyromonas_asaccharolytica, s:Porphyromonas_endodontalis, g:Prevotella, g:Lachnoclostridium* (LDA>3.0, FDR<0.01, **Supplementary Figure S2B**). We performed a stratified analysis of CRC patients based on TNM staging and compared the gut microbiota profiles across different stages. Specifically, patients were grouped into TNM II/III and TNM IV categories, along with a healthy control group. The top 20 most differentially abundant bacterial genera were selected for visualization. As shown in **Supplementary Figure S2C**, genera such as *Ruminococcus, Anaerobacillus, Coprococcus, Clostridium, Anaerobutyricum, Eubacterium*, and *Lachnospira* were significantly reduced in the TNM IV group. In contrast, *Campylobacter, Porphyromonas, Acuticoccus, Klebsiella, Shigella, Escherichia, Lachnoclostridium*, and *Methanococcoides* were markedly enriched in the TNM IV group compared to the other groups.

### 3.2 Differential analysis of gut metabolites

To investigate the differences in metabolic diversity in CRC and H Groups, Alpha diversity analysis was performed. The results showed that the metabolic diversity of the CRC groups was significantly lower than that of the H group (Shannon index, P < 0.01) (**Supplementary Figure S3A**). To further explore the overall differences in metabolite composition among the groups, Beta diversity analysis was conducted to reflect the differences in metabolite distribution. The results revealed significant differences in metabolite distribution among the CRC and H groups (CRC:H PERMANOVA, P = 0.001) (**Supplementary Figure S3B**). Next, we analyzed the differential gut metabolites, the results showed that the contents of Docosapentaenoic acid, LysoPE, Adrenic acid and Indole were increased in the CRC group. The contents of NMS, D-Glucosamine-6-phosphate, Arachidonic acid, 9,10-Epoxyoctadecenoic acid, and D-Galacturonic acid were increased in the H group (P<0.05) (**Supplementary Figure S4**, **Supplementary Table S3**).

### 3.3 Construction of KO genes, metabolites, and KEGG pathway modules related to gut microbiota in CRC and H groups

**Figure 1:**
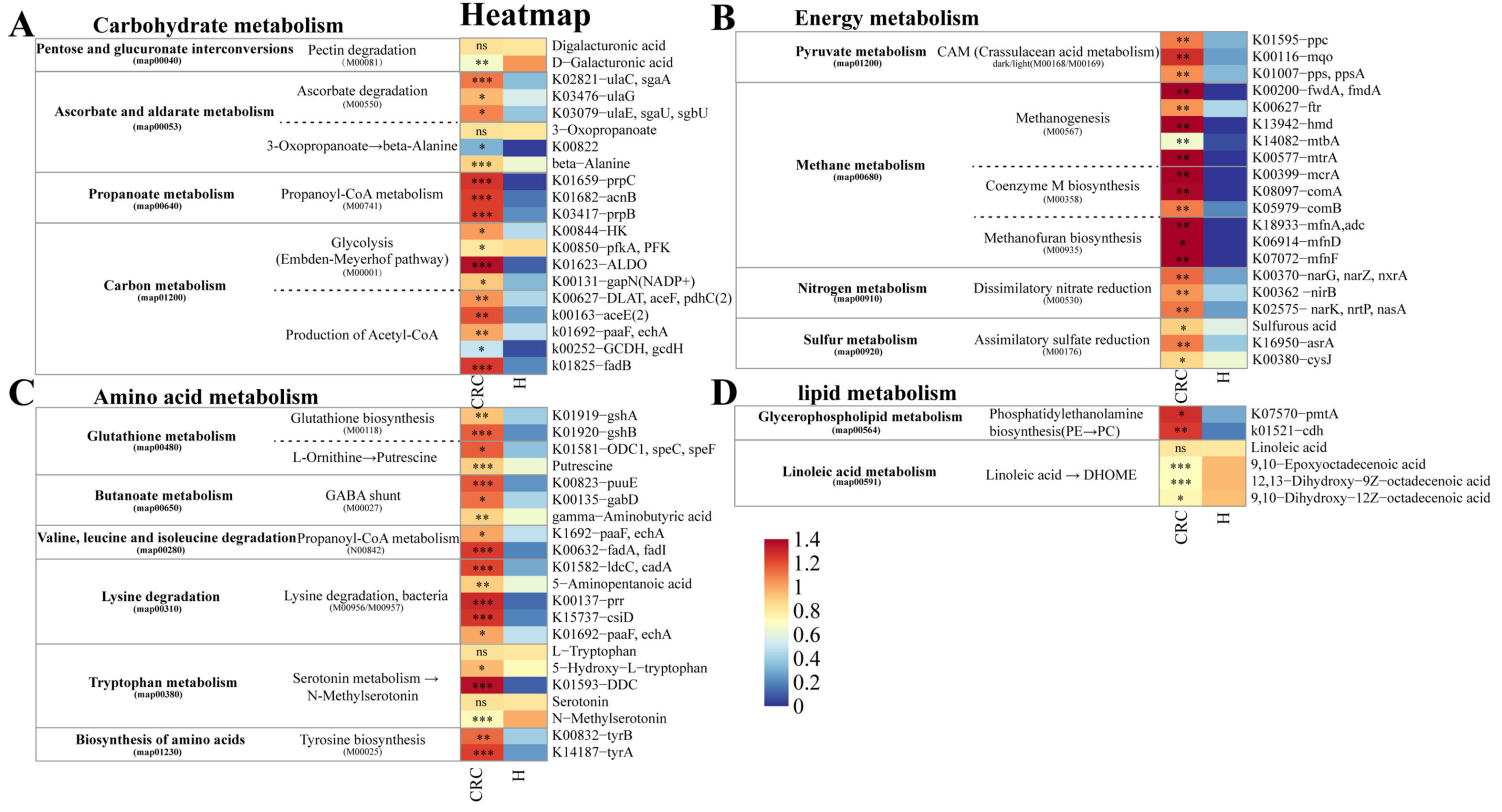
KEGG pathway module for differential KO genes and differential metabolites. **(A–D)** Gene abundances and metabolites content were assessed for significant elevation or depletion in CRC patients compare with the healthy control individuals. **(A)** Relative abundance of KO genes and content of metabolites involved in Carbohydrate metabolism. **(B)** Relative abundance of KO genes and content of metabolites involved in Energy metabolism. **(C)** Relative abundance of KO genes and content of metabolites involved in Amino acid metabolism. **(D)** Relative abundance of KO genes and content of metabolites involved in lipid metabolism. (*P≤ 0.05, **P≤ 0.01, ***P≤ 0.001, ns *P*> 0.05).

To further investigate the metabolic processes of gut microbiota occurring in CRC, we annotated differentially expressed KO genes screened through metagenomics using the KEGG database. We integrated the changes in differentially expressed KO genes and differential metabolites in the KEGG metabolic pathways, and generated heatmaps (**Figures 1A-D**).

In the Carbohydrate metabolism pathway, the majority of differential genes and metabolites were significantly enriched in the CRC groups. For example, Ascorbate degradation (genes: ulaC, ulaG, ulaE), beta-Alanine degradation (gene: K00822, metabolite: beta-Alanine), Propanoyl-CoA metabolism (genes: prpC, acnB, prpB), Glycolysis (genes: HK, pfkA, ALDO, gapN (NADP+)), Production of Acetyl-CoA (genes: DLAT, aceF, pdhC, aceE, paaF, GCDH, gcdH, fadB)) were increased in CRC groups. Additionally, Pectin degradation (metabolite: D-Galacturonic acid) was increased in the H group (**Figure 1A**).

In the Energy metabolism pathway, the majority of differential genes and metabolites were significantly enriched in the CRC groups. For example, CAM (Crassulacean acid metabolism) (genes: ppc, mqo, pps), Methanogenesis (genes: fwdA, ftr, hmd, mtbA, mtrA), Coenzyme M biosynthesis (genes: mcrA, comA, comB), Methanofuran biosynthesis (genes: mfnA, mfnD, mfnF), Dissimilatory nitrate reduction (genes: narG, nirB, narK), Assimilatory sulfate reduction (metabolite: Sulfurous acid, genes: asrA, cysJ), Glutathione biosynthesis (gshA, gshB) were elevated in CRC groups (**Figure 1B**).

In the Amino acid metabolism pathway, the majority of differential genes and metabolites were significantly enriched in the CRC groups. Examples include Production of Putrescine (gene: Putrescine, speC), Gamma-aminobutyric acid (GABA) shunt (genes: puuE, gabD, metabolite: GABA), Propanoyl-CoA metabolism (genes: paaF, fadA), Lysine degradation (genes: ldcC, prr, csiD, paaF, metabolite: 5-Aminopentanoic acid), Tyrosine biosynthesis (genes: tyrB, tyrA), all of which were upregulated in the CRC groups. Furthermore, Tryptophan metabolic pathway (metabolites: L-Tryptophan, 5-Hydroxy-L-tryptophan, Serotonin; gene: DDC) was elevated in CRC groups, while NMS showed decreased abundance (**Figure 1C**).

In the Lipid metabolism pathway, Phosphatidylethanolamine biosynthesis (genes: pmtA, cdh) was significantly upregulated in the CRC groups. Linoleic acid metabolism (metabolites: 9,10-Epoxyoctadecenoic acid, 12,13-Dihydroxy-9Z-octadecenoic acid, 9,10-Dihydroxy-12Z-octadecenoic acid) was increased in the H group (**Figure 1D**).

### 3.4 Bacterial species and metabolites as CRC diagnostic markers

We utilized random forest and logistic regression models to construct classification models for discrimination in CRC patients compared with the healthy control individuals by combining metabolites and microbial data. When five differential bacterial species (*Dialister_pneumosintes, Fusobacterium_sp._oral_taxon_370, Shigella_flexneri, Escherichia_fergusonii, Clostridium_sp._CAG*) were used as biomarkers to distinguish CRC and H group, the AUC was 0.9788 (**Supplementary Figures S5A, B**). When nine differential metabolites (12,13-Dihydroxy-9Z-octadecenoic acid, 5-Aminopentanoic acid, 9,10-Dihydroxy-12Z-octadecenoic acid, Beta-Alanine, Cholesterol, Arachidonic acid, D-Galacturonic acid, NMS, Putrescine) were used as biomarkers to distinguish CRC and H group, the AUC was 0.9740 (**Supplementary Figures S5C, D**). By combining four differential bacterial species (*Fusobacterium_sp._oral_taxon_370, Shigella_flexneri, Escherichia_fergusonii, Clostridium_sp._CAG:12237_41)* and one differential metabolite (NMS) as biomarkers, the AUC could be increased to 0.9940 (**Supplementary Figures S5E, F**).

### 3.5 Correlation analysis of differential microbiota, differential metabolites and differential KO genes

Based on the gut microbiota and metabolite data from patients with CRC and healthy individuals, we carried out a correlation analysis of differential genus-level gut microbiota composition, metabolite expression, and KO gene expression and constructed a network diagram. The results indicated that metabolites that were enriched in the H group, such as NMS, were positively correlated with the abundance of probiotic bacteria like *Lachnospira* and *Eubacterium*, and negatively correlated with potentially carcinogenic bacteria such as *Enterococcus, Escherichia* and *Proteobacteria.* D-galacturonic acid was also positively correlated with the abundance of probiotic bacteria such as *Roseburia* and *Lachnospira*. Metabolites enriched in patients with CRC, such as cholesterol, were positively correlated with potential pathogenic bacteria such as *Escherichia* and *Proteobacteria.* Furthermore, genes involved in methane production (K00200-fwdA, fmdA, K13942-hmd, K00577-mtrA) exhibited a significant positive correlation with *Methanobrevibacter*. *Fusobacterium nucleatum* showed a significant positive correlation with genes involved in acetyl-CoA production (k00252-GCDH, gcdH) (P ≤ 0.05) (**Supplementary Figure S6**).

### 3.6 Construction of NMS metabolic library and targeted detection

Initial metagenomic and untargeted metabolomic analyses revealed significantly higher abundance of B.o and NMS levels in healthy individuals compared with CRC patients (**Supplementary Figures S7A, B**), with a strong positive correlation (P = 1.4×10^-5^) (**Supplementary Figure S7C**). Notably, B.o was identified as the most efficient gut microbiota species for releasing NMS from orange fiber. Consequently, we established a targeted NMS metabolite library to validate NMS production both *in vitro* and *in vivo* (**Supplementary Tables S4**, **S5**).

*In vitro* targeted detection demonstrated significantly increased NMS levels in the culture supernatant of B.o co-cultured with orange fiber (B.o+ orange fiber group), whereas NMS concentrations remained below the detection limit in the Wilkins–Chalgren, B.o alone, and orange fiber alone groups (P<0.0001) (**Supplementary Table S6**). Subsequent *in vivo* animal experiments confirmed that the B.o+orange fiber co-treatment group exhibited high fecal NMS levels, while only trace amounts were detected in the other three groups (P<0.0001) (**Supplementary Table S7**). These results collectively demonstrate the capacity of B.o to metabolize orange fiber into NMS in both experimental systems.

### 3.7 NMS inhibits CRC cell proliferation, cloning, migration, and invasion

**Figure 2:**
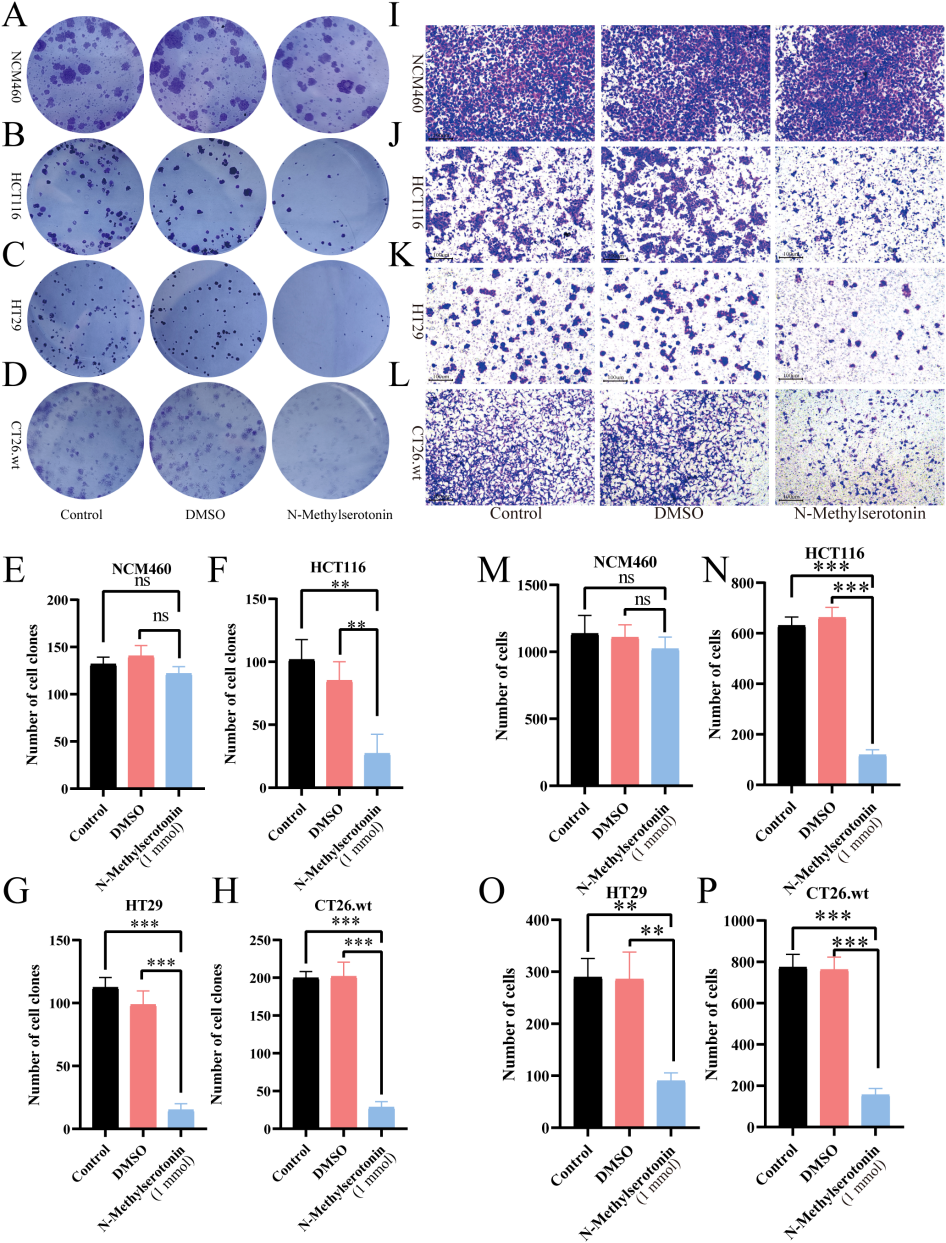
Cloning and transwell formation experiments of NMS. Cloning formation experiment analysis of the effect of NMS on the cloning ability of NCM460 **(A, E)**, HT116 **(B, F)**, HT29 **(C, G)**, and CT26. wt **(D, H)**. Transwell experiment analysis of the effect of NMS on the invasion ability of NCM460 **(I, M)**, HT116 **(J, N)**, HT29 **(K, O)**, and CT26. wt **(L, P)**, (scale bar 100 µm). (*P≤ 0.05, **P≤ 0.01, ***P≤ 0.001, ns *P*> 0.05).

*In vitro* experiments were conducted to investigate the effects of NMS on CRC cells. CCK-8 assay demonstrated that treatment with NMS significantly decreased CRC cell proliferation in a dose-dependent manner (P ≤ 0.05) (**Supplementary Figures S8A-D**). Meanwhile, IC50 values of NCM460 that is inhibited by NMS are 11.11mmol, then by treating equally with NMS, IC50 values of HCT116 are 5.598mmol, IC50 values of HT29 are 5.734 mmol, IC50 values of CT26.wt are 5.015mmol (**Supplementary Figures S8E-H**). Clonogenic assays revealed that treatment with 1 mmol NMS for 48 hours significantly inhibited CRC cell clonogenesis (P ≤ 0.05) (**Figures 2A-H**). Migration assays indicated that NMS suppressed CRC cell migration (P ≤ 0.05) (**Supplementary Figures S9A-D**). Trans-well assays showed that NMS inhibited the invasive capacity of CRC cells (P ≤ 0.05) (**Figures 2I-P**). Importantly, NMS did not affect the biological functions of the normal human epithelial cell line NCM460.

### 3.8 NMS induces CRC cell cycle arrest and promotes apoptosis

**Figure 3:**
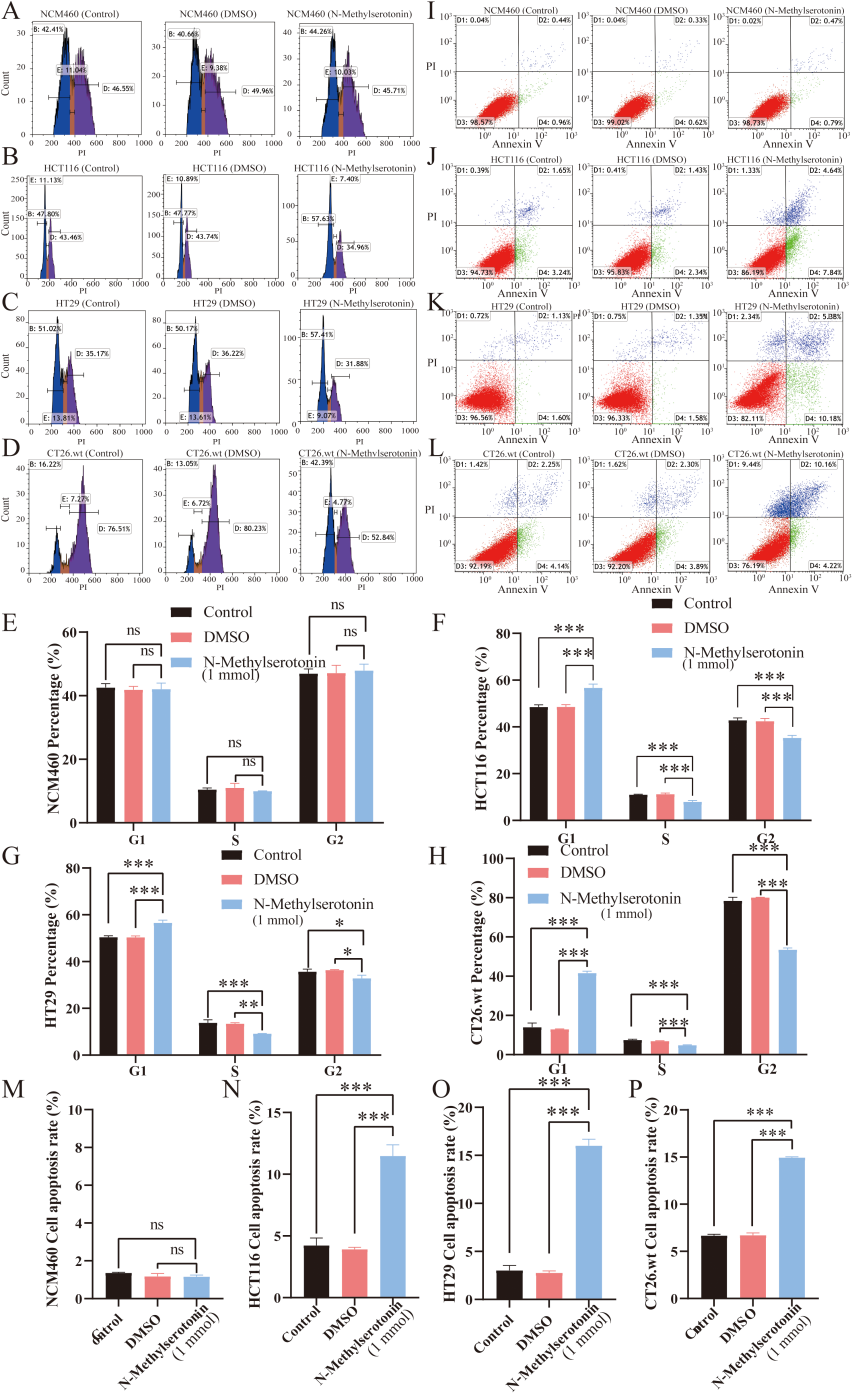
Flow cytometry cell cycle and apoptosis experiments of NMS. After NMS treatment, flow cytometry was used to detect the cell cycle distribution of NCM460 **(A, E)**, HT116 **(B, F)**, HT29 **(C, G)**, and CT26. wt **(D, H)** cells. After NMS treatment, flow cytometry was used to detect the apoptosis rates of NCM460 **(I, M)**, HT116 **(J, N)**, HT29 **(K, O)**, and CT26. wt **(L, P)** cells. (*P≤ 0.05, **P≤ 0.01, ***P≤ 0.001, ns *P*> 0.05).

Flow cytometry was used to analyze the effect of NMS on CRC cell cycle distribution. Following NMS treatment, there was a significant increase in the proportion of CRC cells in the G1 phase, accompanied by decreases in the proportions of cells in the S and G2 phases (P ≤ 0.05), indicative of G1-phase arrest (**Figures 3A-H**). Apoptosis assays revealed that, after 48 hours of treatment with NMS, the proportion of apoptotic cells increased by 6.83% in HCT116, 13.11% in HT29, and 8.25% in CT26.wt CRC cells (P ≤ 0.05) (**Figures 3I-P**). However, NMS had no effect on cell cycle distribution or apoptosis in NCM460 cells.

### 3.9 Therapeutic effects of B.o-derived NMS from orange fiber in AOM/DSS-induced CRC

Preliminary experiments confirmed that intestinal colonization of B.o enables continuous NMS production from orange fiber, which potently inhibits the malignant biological behaviors of CRC cells *in vitro*. To evaluate the *in vivo* antitumor efficacy of B.o, we examined the therapeutic effects of B.o, orange fiber, and NMS in a CRC mouse model.

**Figure 4:**
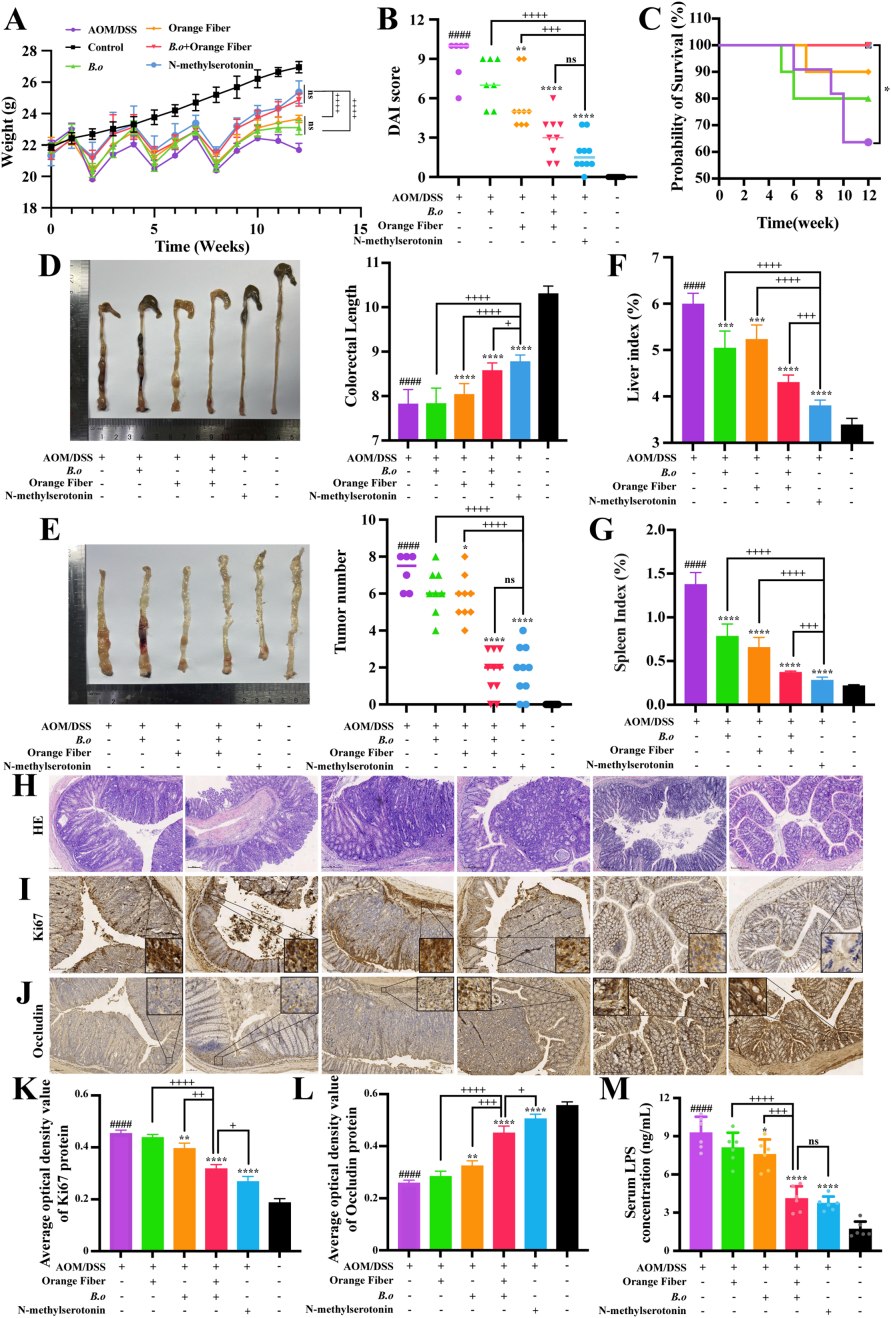
Therapeutic effects of B.o-derived NMS from orange fiber in AOM/DSS-induced CRC. **(A)** Weight changes. **(B)** DAI score. **(C)** survival curve. **(D)** Colorectal length. **(E)** Tumor number. **(F)** Liver index. **(G)** Spleen index. **(H)** HE staining was used to detect histopathological changes in the tissue. **(I)** Immunohistochemical staining of Ki-67. **(J)** Immunohistochemical staining of Occludin. **(K)** The average optical density value of Ki-67 immunohistochemistry. **(L)** The average optical density value of Occludin immunohistochemistry. **(M)** ELISA was used to detect concentration of LPS in mouse serum. (*P≤ 0.05, **P≤ 0.01, ***P≤ 0.001, ns *P*> 0.05).

During modeling, both B.o+orange fiber (BF) and NMS (N) treatments significantly ameliorated weight loss compared with single treatments with either orange fiber (F) or B.o (B) (**Figure 4A**). The F, BF, and N treatments markedly reduced disease activity index (DAI) scores (P<0.01) (**Figure 4B**). Compared with AOM/DSS controls, both BF and N treatments significantly decreased mortality (P<0.05) with comparable efficacy (**Figure 4C**). AOM/DSS treatment significantly shortened colorectal length versus the controls, whereas F, BF, and N treatments effectively restored intestinal length, with NMS showing superior protection (**Figure 4D**). Tumor burden analysis revealed the most significant reduction in the N and BF groups (**Figure 4E**). Furthermore, elevated hepatosplenic indices in AOM/DSS mice were significantly attenuated by all treatments, particularly by NMS (**Figures 4F, G**).

Histopathological examination (H&E staining) showed colorectal tumors in the AOM/DSS, B, and F groups, whereas the BF and N groups predominantly exhibited intramucosal carcinoma or high-grade intraepithelial neoplasia, with some individuals showing only epithelial hyperplasia (**Figure 4H**). Immunohistochemical analysis demonstrated that NMS most effectively suppressed AOM/DSS-induced Ki-67 overexpression (**Figures 4I, K**) while significantly upregulating occludin expression (**Figures 4J, L**). NMS treatment also significantly reduced serum LPS levels, indicating improved gut barrier function (**Figure 4M**).

In summary, NMS effectively inhibited AOM/DSS-induced CRC progression by reducing malignancy, regulating intestinal permeability, and maintaining epithelial barrier integrity. Importantly, B.o combined with orange fiber also demonstrated significant anti-CRC effects.

### 3.10 Target prediction and mechanistic validation of NMS in CRC inhibition

**Figure 5:**
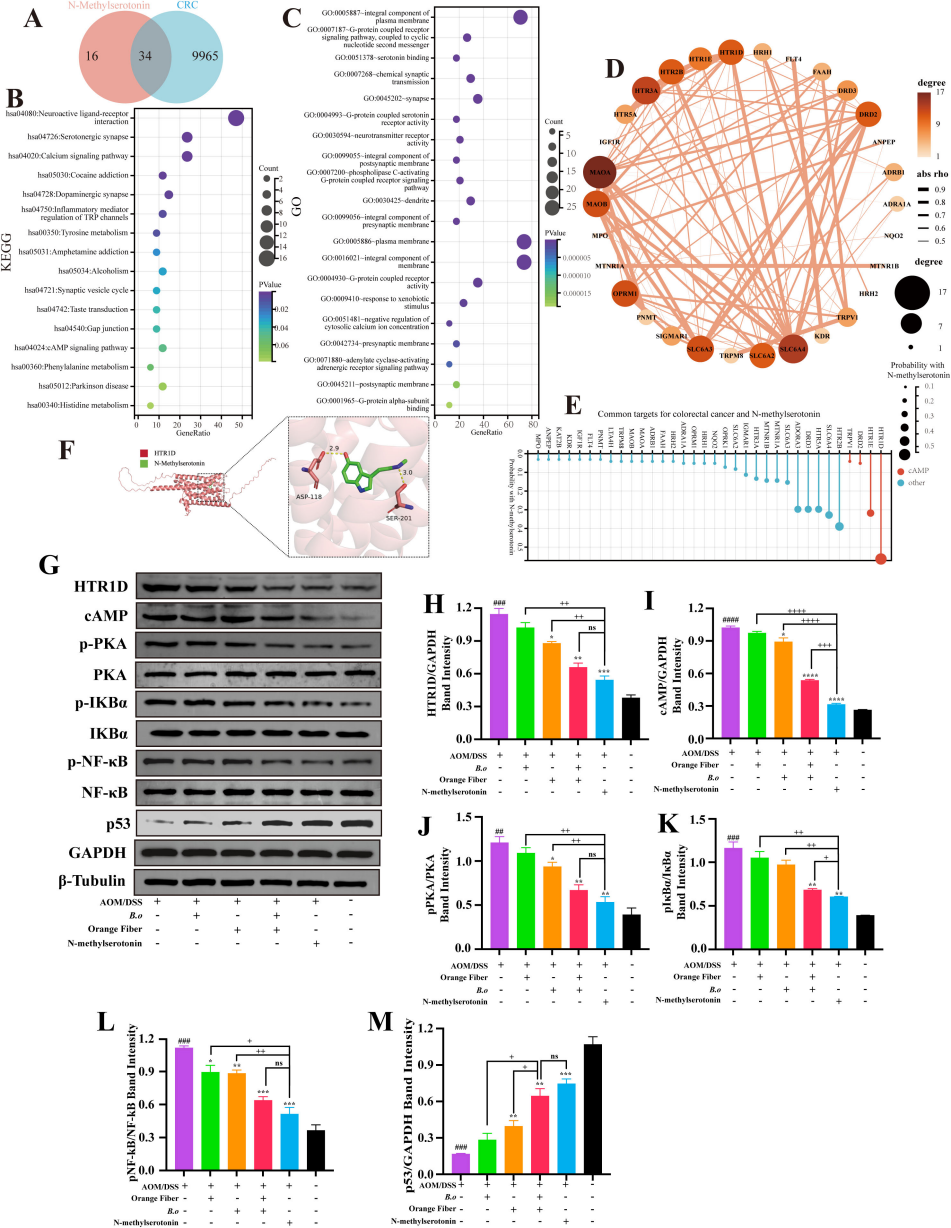
Network pharmacology and experimental validation of NMS inhibit colorectal cancer. **(A)** Cross Venn diagram of NMS related targets and CRC related targets. **(B)** GO and **(C)** KEGG enrichment analysis bubble plots. **(D)** Correlation network diagram. **(E)** The lollipop chart shows a correlation between 34 targets and NMS. **(F)** Molecular docking model of NMS with HTR1D. **(G)** Western blot analysis of key protein expression. **(H-M)** Quantitative analysis of the protein levels from. (*P≤ 0.05, **P≤ 0.01, ***P≤ 0.001, ns *P*> 0.05).

Previous experiments confirmed that NMS produced by B.o from orange fiber can inhibit CRC development both *in vitro* and *in vivo*, but its targets and mechanisms remained unknown. Therefore, we used network pharmacology and molecular docking to predict its targets, followed by western blot validation. First, we identified 50 potential NMS targets from the Swiss Target Prediction database and 9965 known CRC-related targets from GeneCards, with 34 overlapping targets (**Figure 5A**). GO analysis suggested NMS may be involved in serotonin binding and G-protein coupled serotonin receptor activity (**Figure 5B**). KEGG analysis indicated that NMS might participate in the serotonergic synapse and cyclic adenosine monophosphate (cAMP) signaling pathway (**Figure 5C**). A network diagram displayed potential targets of NMS for CRC treatment (**Figure 5D**). The lollipop plot results showed that 5-hydroxytryptamine receptor 1D (HTR1D) in the cAMP signaling pathway might be a key target for NMS in CRC treatment (**Figure 5E**). Molecular docking between HTR1D and NMS showed high compatibility with binding energy of -6.4 kcal/mol, indicating a direct interaction (**Figure 5F**).

We then analyzed the protein levels of HTR1D, cAMP, phosphorylated protein kinase A (p-PKA), phosphorylated nuclear factor of kappa light polypeptide gene enhancer in B-cells inhibitor, alpha (p-IκBα), phosphorylated nuclear factor kappa-light-chain-enhancer of activated B cells (p-NF-κB) by western blotting. AOM/DSS-induced CRC mice showed significantly increased HTR1D, cAMP, and p-PKA protein levels in colorectal tissues. After treatment, HTR1D, cAMP, and p-PKA levels were significantly lower in the orange fiber, combination therapy, and NMS groups compared with the AOM/DSS group. Combination therapy showed more pronounced inhibition than single treatments. Moreover, HTR1D and PKA protein expression in the combination group showed no significant difference from the NMS group. p-IκBα expression in the AOM/DSS group was slightly reduced by single treatment but significantly inhibited by combination and NMS treatments, with the NMS group showing stronger inhibition than the combination group. Single treatments, combination therapy, and NMS treatment all significantly inhibited NF-κB phosphorylation. Analysis showed that IκBα and NF-κB activation in the combination group was significantly lower than in the single treatment groups, with NF-κB phosphorylation showing no significant difference from the NMS group (**Figures 5G-L**).

These experiments revealed that NMS produced by the B.o and orange fiber combination mainly inhibits CRC through the HTR1D/cAMP/PKA/IκBα/NF-κB pathway. To further investigate its mechanism, we performed western blot detection of downstream cytokines (**Figures 5G, M**). The results showed significantly decreased tumor protein p53 (p53) expression. p53 protein levels were significantly lower in the orange fiber, combination, and NMS groups compared with the CRC group, with the combination group showing significantly higher inhibition than the single treatment groups, and NMS showing similar inhibition to the combination group.

In summary, NMS produced by B.o from orange fiber downregulates HTR1D expression, inhibits cAMP/PKA/IκBα/NF-κB pathway activation, reverses AOM/DSS-induced p53 reduction.

### 3.11 Differences in gut microbiota and metabolites among treatment groups based on 16S rRNA sequencing and untargeted metabolomics

**Figure 6:**
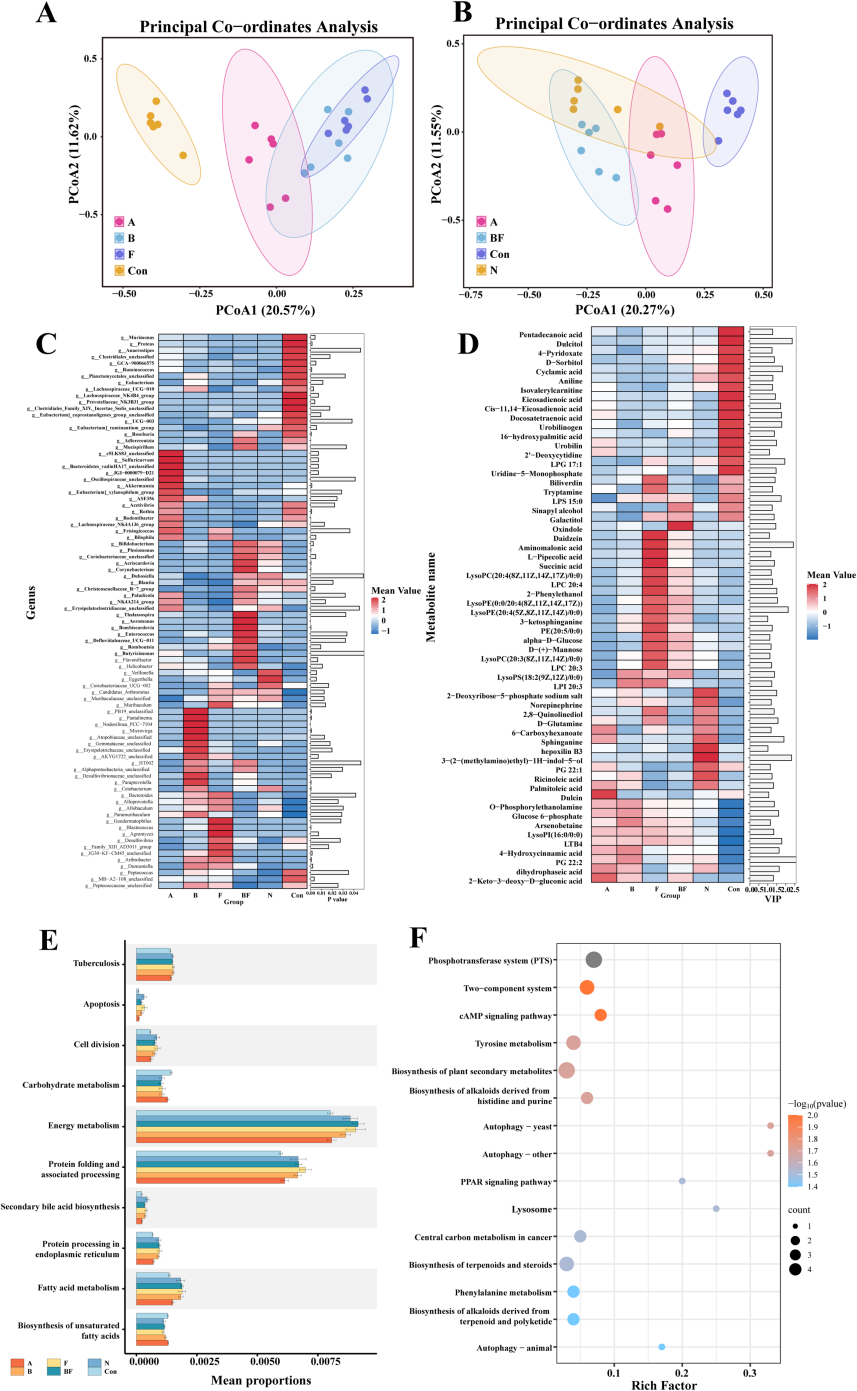
16sRNA and untargeted metabolomics sequencing of mouse feces. **(A)** PCOA analysis of mouse gut microbiota in AOM/DSS group (A group), B.o group (B group), Orange fiber group (F group), and Control group (Con group). **(B)** PCOA analysis of mouse metabolite in AOM/DSS group (A group), B.o+orange fiber group (BF group), Control group (Con group), and NMS group (N group). **(C)** Differential microbial expression heatmap. **(D)** Differential metabolite expression heatmap. **(E)** PICRUSt2 predicts KEGG functional stamp map of gut microbiota. **(F)** KEGG enrichment analysis bubble plot of differential metabolites.

We collected fecal samples from each group for 16S rRNA sequencing and untargeted metabolomics. Beta diversity analysis showed clear separation between the F and BF groups and the A group (**Figures 6A, B**). For differential analysis of the gut microbiota, we excluded B.o from the analysis because of prior administration. The results showed increased abundance of *g:Lachnospiraceae_NK4B4_group*, *g:Murimonas* in the Con group; *g:Oscillospiraceae_unclassified*, *g:Bilophila* in the A group; *g:Aeriscardovia*, *g:Bifidobacterium* in the BF and N groups; *g:Eubacterium_xylanophilum_group* in the N and Con groups; *g:Blautia*, *g:Christensenellaceae_R−7_group* in the BF, N, and Con groups; and *g:Candidatus_Arthromitus* in the F, BF, and N groups (P<0.05) (**Figure 6C**). For metabolomic analysis, we excluded NMS because of prior administration. The results showed an increase in arsenobetaine and LTB4 in the A group; 16-hydroxypalmitic acid, dulcitol, and pentadecanoic acid in the Con group; oxindole in the BF group; ricinoleic acid in the N group; and L-pipecolic acid in the F group (P<0.05) (**Figure 6D**).

We conducted KEGG enrichment analysis using the differential microbial communities and metabolites among the groups. The KEGG analysis results of differential microbial communities include apoptosis, cell division, carbohydrate metabolism, energy metabolism, protein folding and associated processing, secondary bile acid biosynthesis, protein processing in endoplasmic reticulum, fatty acid metabolism, and biosynthesis of unsaturated fatty acids (P<0.05) (**Figure 6E**). The results of differential metabolite enrichment analysis mainly include phosphotransferase system (PTS), two-component system, cAMP signaling pathway, biosynthesis of plant secondary metabolites, biosynthesis of histidine and purine alkaloids derived from history and purify, tyrosine metabolism, central carbon metabolism in cancer, phenylalanine metabolism, terpenoid and steroid biosynthesis. of terpenoids and steroids (P<0.05) (**Figure 6F**).

By integrating sequencing data of gut microbiota and metabolites from each treatment group, we performed correlation analysis between differentially abundant bacterial genera and metabolites and generated heatmaps. The results demonstrated that in Group A, enriched metabolites such as leukotriene B4 showed significant negative correlations with probiotic abundances including *g:Roseburia*, *g:Akkermansia*, *g:Lachnospiraceae_NK4B4_group*, *g:Murimonas*, and *g:Mucispirillum*. Arsenobetaine exhibited positive correlations with potentially carcinogenic bacteria including *g:Oscillospiraceae_unclassified* and *g:Helicobacter*, while showing negative correlations with probiotic genera such as *g:Eubacterium* (P<0.05).In the Con group, enriched metabolites including 16-hydroxypalmitic acid displayed positive correlations with probiotics *g:Lachnospiraceae_NK4A136_group* and *g:Blautia*, but negative correlations with potentially pathogenic bacteria *g:Allobaculum* (P<0.05). Pentadecanoic acid showed significant positive correlations with probiotic bacteria *g:Eubacterium_xylanophilum_group* (P<0.05) (**Supplementary Figure S10**). In conclusion, changes in intestinal metabolites among treatment groups may correlate with gut microbiota alterations. Increased beneficial metabolites may associate with elevated probiotics, while increased potential pathogenic metabolites may relate to increased potential pathogens.

## 4 Discussion

In the analysis of gut microbiota and metabolites in this study, no significant differences were observed in alpha diversity indices (Chao1 and Shannon) between the CRC group and the healthy control (H) group, whereas beta diversity indices (based on PERMANOVA analysis) showed significant differences. The results of alpha diversity indicate that the overall species number and distribution uniformity of the gut microbial communities were similar between CRC patients and healthy individuals. However, the beta diversity analysis revealed that although the overall diversity levels were comparable between the two groups, there were fundamental differences in both specific species of bacterial constituting these communities and their relative abundances. This pattern—absence of alpha diversity differences alongside significant beta diversity differences—has also been reported in microbiome studies of colorectal cancer and other diseases (18–20). This further suggests that the onset and progression of CRC are not associated with a “total collapse” of the gut microbiota, but are closely linked to dysbiosis of specific microbial taxa.

Gut microbiota components and their metabolites have been extensively studied for their impact on CRC (21). Numerous studies have noted a significant decrease in Lactobacillus abundance in patients with CRC (22, 23). Treatment with Lactobacillus capsules has been shown to significantly reduce the incidence of postoperative pneumonia, surgical site infections, and anastomotic leakage, as well as shorten time to discharge, in patients with CRC (24). Akkermansia inhibits CRC by specifically inhibiting the tryptophan-mediated AhR/β-catenin signaling pathway and promotes M1 macrophage polarization via NOD-like receptor thermal protein domain associated protein 3 (NLRP3)-dependent mechanisms both *in vivo* and *in vitro* (25, 26). Studies have demonstrated that Lachnospira suppresses CRC occurrence and development; indeed, Lachnospira abundance negatively correlates with CRC cell proliferation, and its metabolites inhibit CRC cell proliferation (27, 28). Treating a mouse model of CRC with NMS resulted in decreased abundance of Bacteroides, Parabacteroides, Enterococcus, and increased abundance of probiotic bacteria such as Lactococcus, Akkermansia, and Lachnospiraceae_NK4A136_group.

Conjoint analysis revealed that genes involved in glycolysis (HK, pfkA, PFK, ALDO, gapN (NADP+)) and acetyl-CoA biosynthesis (DLAT, aceF, pdhC, aceE, paaF, GCDH, gcdH, fadB) were upregulated in the CRC groups. Fusobacterium nucleatum abundance showed a significant positive correlation with the expression of genes involved in acetyl-CoA synthesis (k00252-GCDH, gcdH). During tumorigenesis, the glycolysis pathway is significantly activated, leading to increased glucose uptake by tumor cells, enhanced acetyl-CoA generation, and lactate accumulation, thus promoting tumor cell survival (29, 30). Jie et al. demonstrated that Fusobacterium nucleatum abundance in CRC is positively correlated with glucose metabolism and promotes glycolysis through upregulation of enolase 1 expression (31). Methanobrevibacter abundance was increased, and methane metabolism genes (fwdA, ftr, hmd, mtbA, mtrA) were significantly upregulated, and these two phenomena exhibited a significant positive correlation. Previous studies have indicated that methane production is not influenced by substrates but depends solely on Methanobrevibacter abundance, suggesting an association between alterations in methane metabolism pathways and Methanobrevibacter (32). We observed a significant increase in the concentrations of various linoleic acid metabolite components of in the lipid metabolism pathway in healthy individuals. Studies have shown that linoleic acid can promote tumor cell apoptosis, as well as inhibit tumor angiogenesis and proliferation (33, 34). Increased linoleic acid metabolism is associated with elevated abundance of probiotic bacteria such as Rothia, Streptococcus, and Lactobacillus (35–37). In summary, compared with healthy individuals, metabolic pathways in patients with CRC patients were significantly altered, glycolysis, methane energy metabolism, beneficial amino acid degradation, NMS production, and linoleic acid metabolism were significantly increased. These changes can be attributed to dysbiosis of the gut microbiota, with an increase in pathogenic bacteria and a decrease in probiotic bacteria observed in patients with CRC.

Studies have shown that B.o, as a symbiotic bacterium capable of efficiently utilizing plant and host glycans (38, 39), can significantly improve intestinal barrier function and alleviate insulin resistance through the metabolism of glucomannan (40). Notably, increased abundance of B.o is positively correlated with intestinal secretory immunoglobulin A (IgA) levels, and this immunomodulatory effect effectively inhibits the penetration of pathogens and toxins into the intestinal epithelium, thereby maintaining intestinal homeostasis (41) and demonstrating significant therapeutic effects in mouse colitis models (42). From a metabolic perspective, the interaction between B.o and orange fiber can produce NMS with multiple bioactive functions. Serotonin, a multifunctional neurotransmitter synthesized by enterochromaffin cells, plays a key role in cell growth regulation and signal transduction (43). NMS is generated through catalysis by indolethylamine-N-methyltransferase (44). NMS not only blocks serotonin reuptake via the 5-hydroxytryptamine receptor 7 (5-HTR7)-mediated signaling pathway (45), but more importantly, as a specific metabolite produced by B.o from orange fiber, it exhibits significant metabolic regulatory functions, including improving obesity phenotypes, regulating hepatic glycogen synthesis, accelerating intestinal transit, and coordinating the expression of circadian rhythm genes in the liver and colon (9). Clinical studies further confirm a dose-dependent positive correlation between orange fiber intake and fecal NMS levels, with this metabolite showing significant associations with carbohydrate-active enzymes (such as rhamnogalacturonan lyase and β-glucan/cellulase) (46–48). This study found that NMS treatment and the combination of B.o with orange fiber effectively prevented weight loss and increased DAI scores during AOM/DSS modeling, reduced CRC tumor numbers, restored colorectal length, and alleviated hepatosplenomegaly in CRC model mice. These findings are consistent with previous studies showing that intestinal probiotics and their metabolites can effectively improve the typical pathological features of CRC (such as colon shortening, tumorigenesis, and hematochezia). Oral lactulose administration can reduce splenomegaly in AOM/DSS-induced CRC model mice (48). Ma et al. demonstrated that gavage administration of *Lactobacillus plantarum* 12 alleviated splenomegaly and thymic atrophy in AOM/DSS-induced CRC model mice (49). Particularly noteworthy is the unique immunomodulatory properties of NMS: on one hand, it inhibits tumor malignant progression by downregulating the proliferation marker Ki-67; on the other hand, it improves intestinal barrier function by enhancing the expression of tight junction protein Occludin and reducing LPS levels. Additionally, this metabolite can regulate the expression of circadian rhythm and fatty acid metabolism-related genes (9), providing a molecular basis for its multi-organ protective effects. In summary, while current research on the gut microbiome and colorectal cancer has predominantly focused on metabolites such as short-chain fatty acids (50), beneficial amino acids (51), cholesterol (52), and genotoxins derived from pathogenic bacteria, the role of microbially derived N-methylserotonin (NMS) has remained largely unexplored in this context. Existing studies on NMS have primarily focused on chemical, metabolic, or neurological settings (9, 44, 53), which results that its potential role in cancer biology has not been unaddressed. In contrast, our work reveals a previously unrecognized microbial metabolic pathway and its mechanistic significance in suppressing CRC. We demonstrate for the first time that Bacteroides ovatus (B.o) can metabolize orange fiber to produce NMS, which exerts a protective effect against colorectal carcinogenesis. Moreover, we identify a novel signaling axis through which NMS may downregulate HTR1D expression and subsequently inhibits the cAMP/PKA/IκBα/NF-κB pathway, ultimately suppressing tumor proliferation and promoting apoptosis.

This study confirmed that NMS treatment and the combination of B.o with orange fiber may exert anti-CRC effects by downregulating HTR1D expression, inhibiting the cAMP/PKA/IκBα/NF-κB pathway, and regulating the expression of downstream cytokines p53. Serotonin receptors (HTRs) are a class of membrane protein receptors that recognize the neurotransmitter serotonin and are widely distributed in the central nervous system and peripheral tissues (32), regulating various physiological processes including mood, cognition, behavior, sleep, pain perception, cardiovascular and intestinal motility, as well as immune responses (54). Studies have shown that HTR1D is highly expressed in CRC cells and is associated with poor prognosis (55). cAMP as a key secondary messenger, mainly regulates the transcriptional activity of multiple downstream target genes by binding to and activating the regulatory subunit of PKA. Notably, PKA can also participate in the precise regulation of cell signal transduction by phosphorylating kinases such as raf-1 proto-oncogene, serine/threonine kinase, glycogen synthase kinase 3, and IκB inhibitory protein α. During CRC development, abnormal activation of the cAMP/PKA signaling pathway in colonic epithelial cells significantly upregulates β-catenin signaling, thereby promoting tumor progression (56). Clinical sample analysis shows that in CRC tissues, the mRNA and protein levels of protein kinase A, regulatory subunit type I alpha (PKARIα) and a-kinase anchoring protein 10 (AKAP10) are significantly increased and positively correlated with invasion depth, differentiation degree, and poor prognosis (57). Mechanistically, PKA activation induces IκB phosphorylation, promoting its ubiquitination and degradation, ultimately leading to nuclear translocation of NF-κB and its binding to specific DNA sequences to activate downstream target gene transcription (58). Under physiological conditions, NF-κB dimers typically exist in an inactive form (59) and are retained in the cytoplasm through interaction with IκBα. When IκBα is phosphorylated and subsequently ubiquitinated and degraded, the activated NF-κB dimer can translocate into the nucleus, bind to corresponding DNA sequences, and initiate target gene transcription (58). Numerous studies have shown that abnormal activation of the NF-κB signaling pathway promotes CRC proliferation, metastasis, angiogenesis, impairs the efficacy of CRC chemotherapy drugs, and facilitates CRC development. In terms of therapeutic strategies, ginsenoside Rh4 inhibits CRC in a gut microbiota-dependent manner by regulating microbiota-mediated bile acid metabolism and promoting ursodeoxycholic acid production to modulate the NF-κB signaling pathway (60). Similarly, L. plantarum improves CRC by producing conjugated linoleic acid to inhibit the NF-κB pathway and downstream pro-inflammatory cytokines (61) The study by Yue et al. showed that *Bifidobacterium longum* SX-1326 ameliorated gastrointestinal toxicity induced by irinotecan chemotherapy by regulating the p53 signaling pathway and the gut–brain axis. When p53 function is impaired or lost, DNA damage accumulates in cells, cell cycle control is disrupted, and cancer development is promoted (62). Currently, several anticancer drugs targeting the B-cell lymphoma-2/Bcl-2-associated X protein (Bcl-2/Bax) pathway have been approved or are under investigation in clinical trials. Related studies have also confirmed that overexpression of Bcl-2 significantly inhibits p53-induced apoptosis (63).

## 5 Limitations

This study has several limitations that should be acknowledged. First, the sample size of human fecal specimens is relatively small, which may affect the generalizability of our findings. Second, the cohort mostly consisted of patients with advanced TNM stages (III and IV), and this imbalance limits our ability to assess whether the decrease in B. ovatus and NMS is associated with disease progression. In future research, we aim to establish cohorts that are larger, multi-center and longitudinal, and have more balanced patient composition coming from all TNM stages. Finally, although our molecular docking and protein blot data strongly suggest the involvement of the HTR1D/cAMP/PKA/NF-κB axis, further mechanistic validation—such as HTR1D overexpression or knockdown experiments, or the use of specific agonists or antagonists—is required to establish causality. Addressing these issues is a major objective of our ongoing and future research. Despite these limitations, this study provides the first evidence linking B. ovatus-derived NMS to the suppression of CRC, offering a novel and promising metabolite target for CRC treatment strategies.

## 6 Conclusion

This study reveals the first time crucial role of B.o and its metabolite NMS in the prevention and treatment of CRC. CRC patients exhibited significantly reduced levels of B.o and NMS in the gut, which were positively correlated. Experimental results demonstrated that B.o metabolizes orange fiber to produce NMS, which effectively inhibits CRC cell proliferation and migration, and induces apoptosis. In the AOM/DSS-induced mouse model, treatment with B.o combined with orange fiber or NMS alone reduced tumorigenesis and improved intestinal barrier function. Mechanistic studies revealed that these effects could be mediated through downregulation of HTR1D expression, inhibition of the cAMP/PKA/IκBα/NF-κB signaling pathway, and modulation of p53 and Bcl-2/Bax expression. Additionally, this therapeutic approach optimized gut microbiota composition and metabolite profiles. These findings not only elucidate the antitumor mechanism of the B.o/NMS system but also provide novel insights into gut microbiota-based strategies for CRC treatment.

## Data Availability

The datasets presented in this study can be found in online repositories. The names of the repository/repositories and accession number(s) can be found below: https://www.ncbi.nlm.nih.gov/, PRJNA1138893.
