# Supplementary material for: Bacteroides ovatus-derived N-methylserotonin inhibit colorectal cancer via the HTR1D-mediated cAMP-PKA-NF-κB signaling axis
Source: Front Immunol. 2025 Nov 24;16:1696701. doi: 10.3389/fimmu.2025.1696701 (PMC12682799; doi:10.3389/fimmu.2025.1696701)
Supplement: Supplementary file 1 [file Table1.docx]

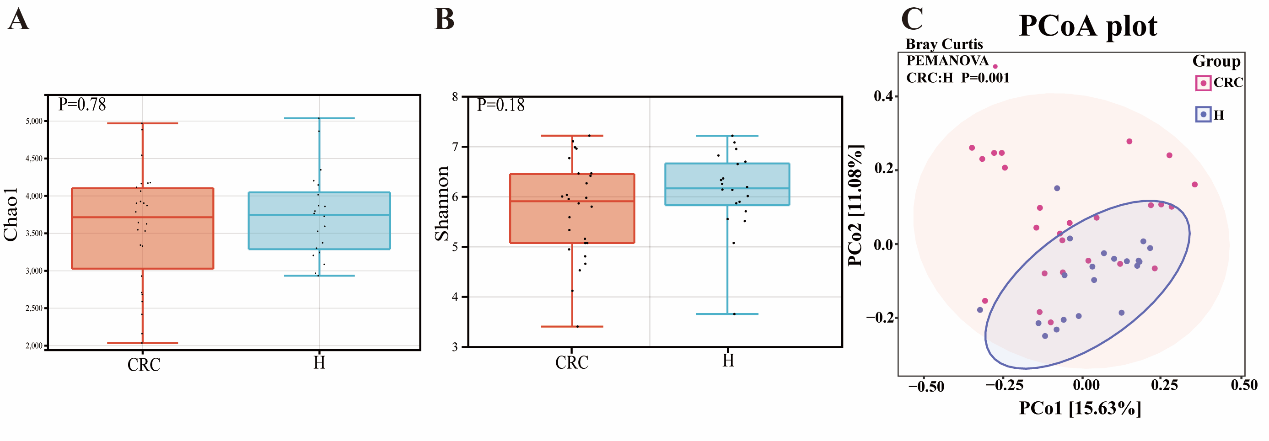


Supplementary Figure 1: Alpha and Beta diversity analysis of gut microbiota.

(A) Box plot of gut microbiota alpha diversity at the species level estimated by the Chao1 index. (B) Box plot of gut microbiota alpha diversity at the species level estimated by the Shannon index. (C) Principal coordinates analysis (PCoA) with Bray-Curtis distance plots represent gut microbiota beta diversity.


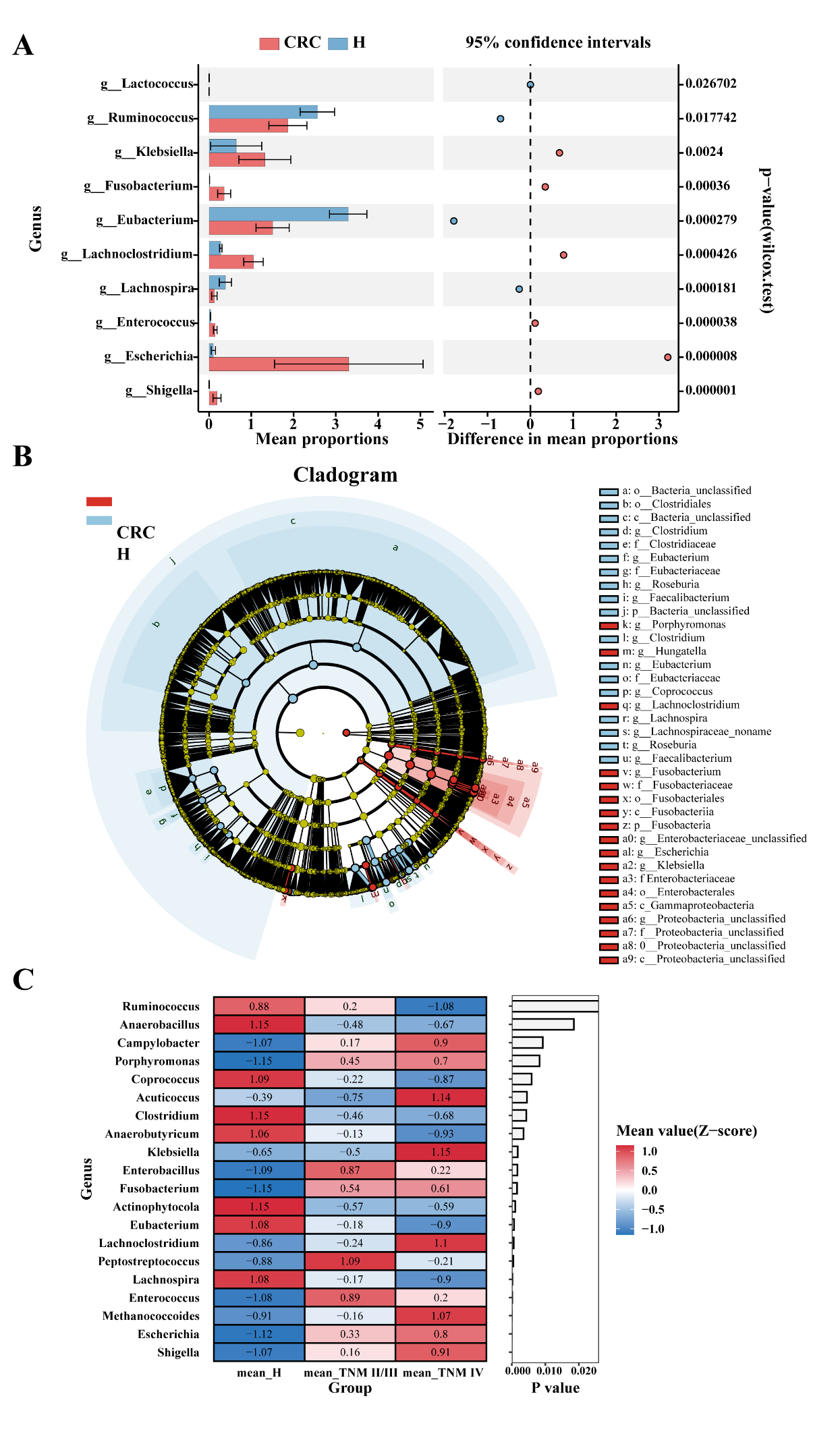


Supplementary Figure 2: Differential analysis of gut microbiota.

(A) Differences in the abundance of gut microbiota between CRC patients compared to healthy controls. (B) The branching diagram shows the LEfSe LDA analysis of gut microbiota in CRC patients compared to healthy controls (LDA>3.0, FDR<0.01). (A) Differential expression heatmap of gut microbiota in CRC patients among healthy controls group, TNM Ⅱ/Ⅲ group and TNM Ⅳ group


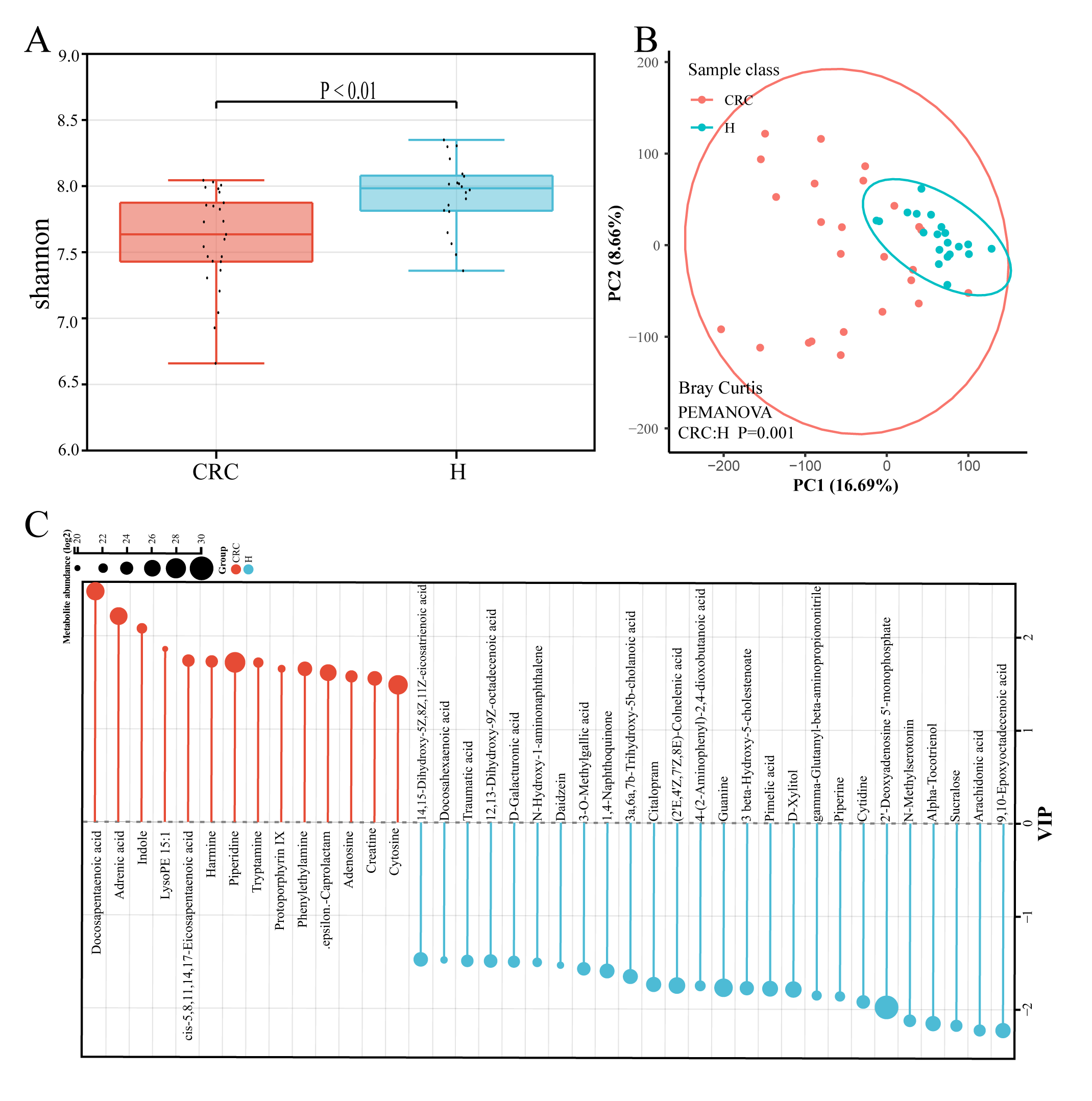


Supplementary Figure 3: Differential analysis of gut metabolites.

(A) Box plot of gut metabolites alpha diversity at the content level estimated by the Shannon index. (B) Principal coordinates analysis (PCoA) with Bray-Curtis distance plots represent gut metabolites beta diversity.


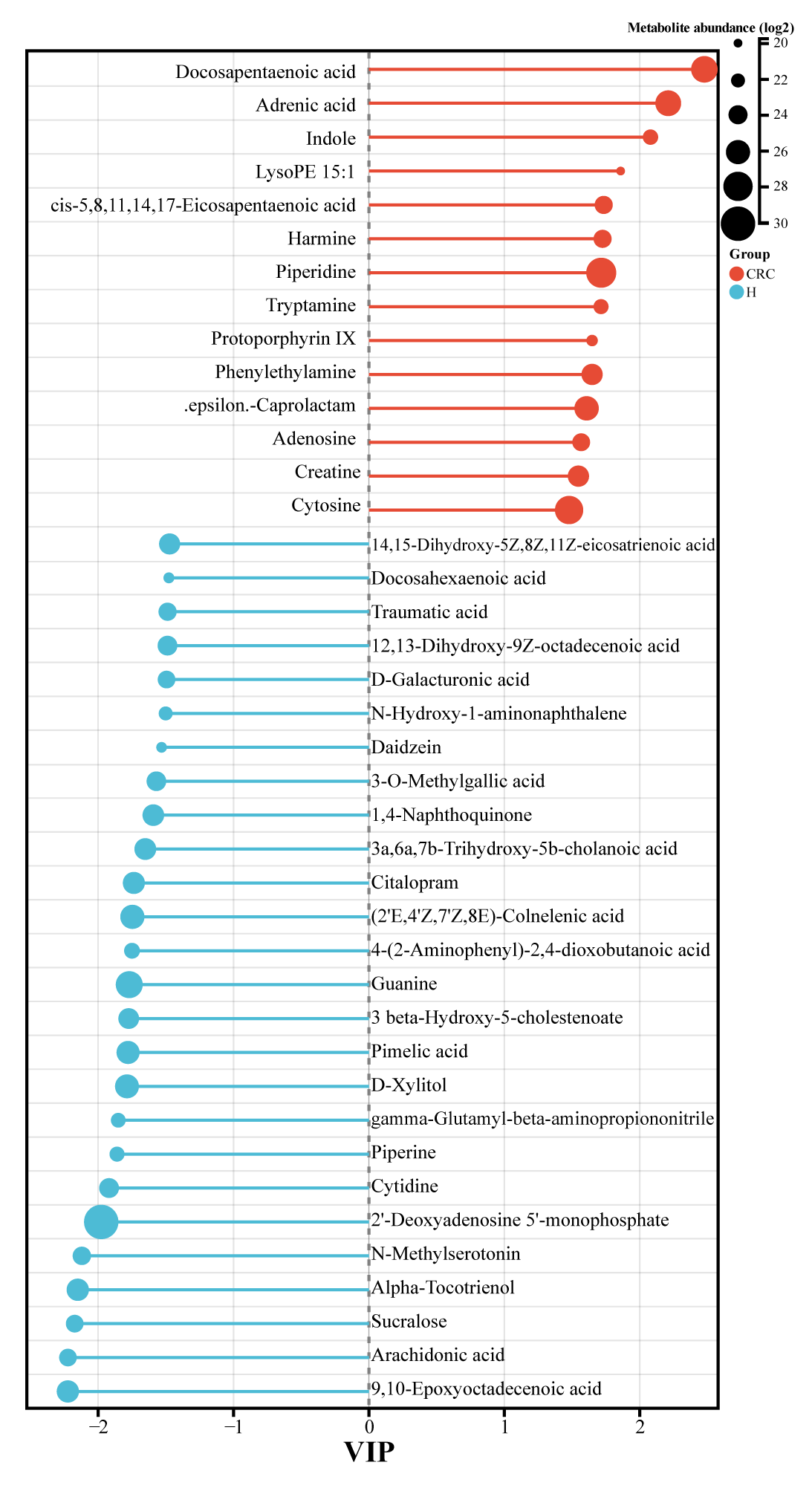


Supplementary Figure 4: Lollipop Chartp showing the differential content of gut metabolites in CRC patients compare with the healthy control individuals. Screen metabolites with |VIP|>1, and sort them by VIP value.


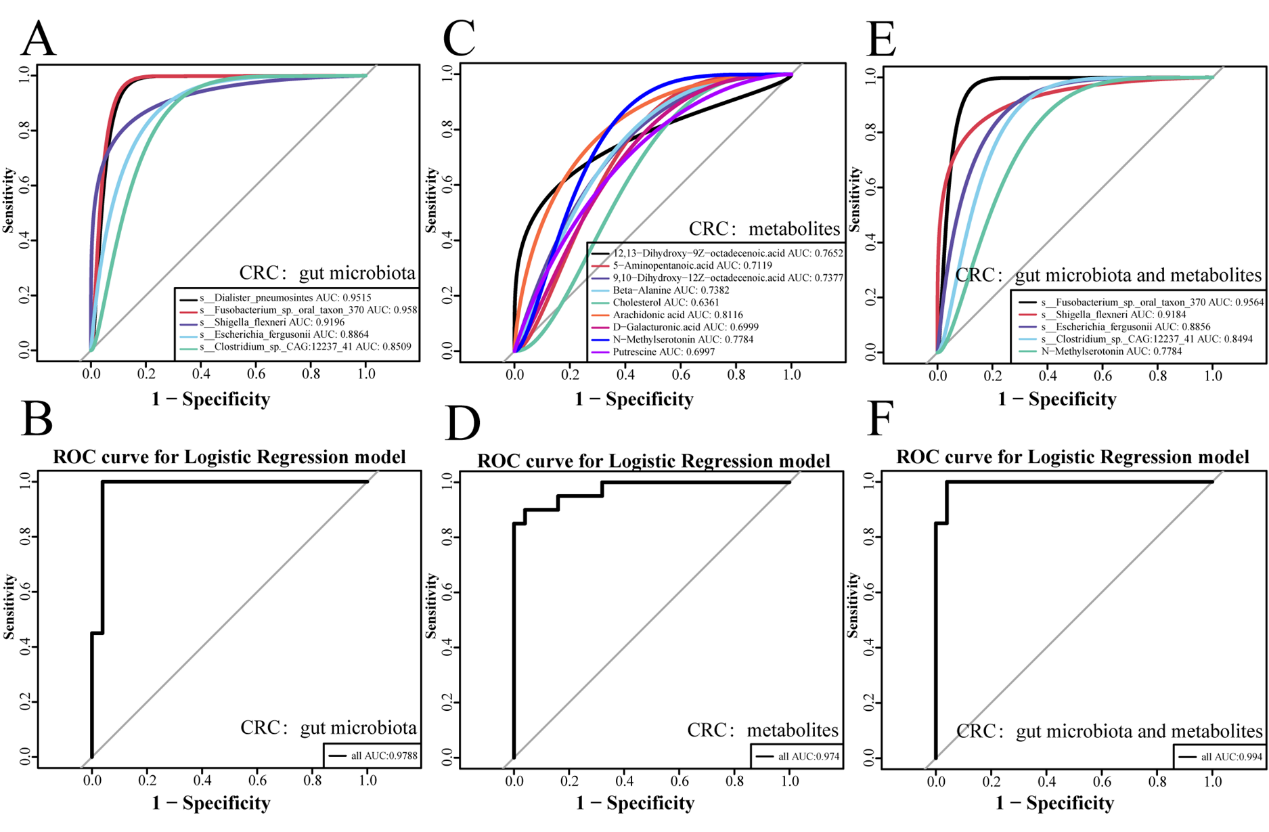


Supplementary Figure 5: Constructing a diagnostic model using random forest screening for differential microbiota and metabolites.

(A-B) Five differential microbiota were screened to distinguish CRC and H, with AUC=0.9788. (C-D) Screen 9 differential metabolites to distinguish CRC and H, AUC=0.974. (E-F) Four differential microbiota and one differential metabolite were screened to distinguish CRC and H, with AUC=0.994.


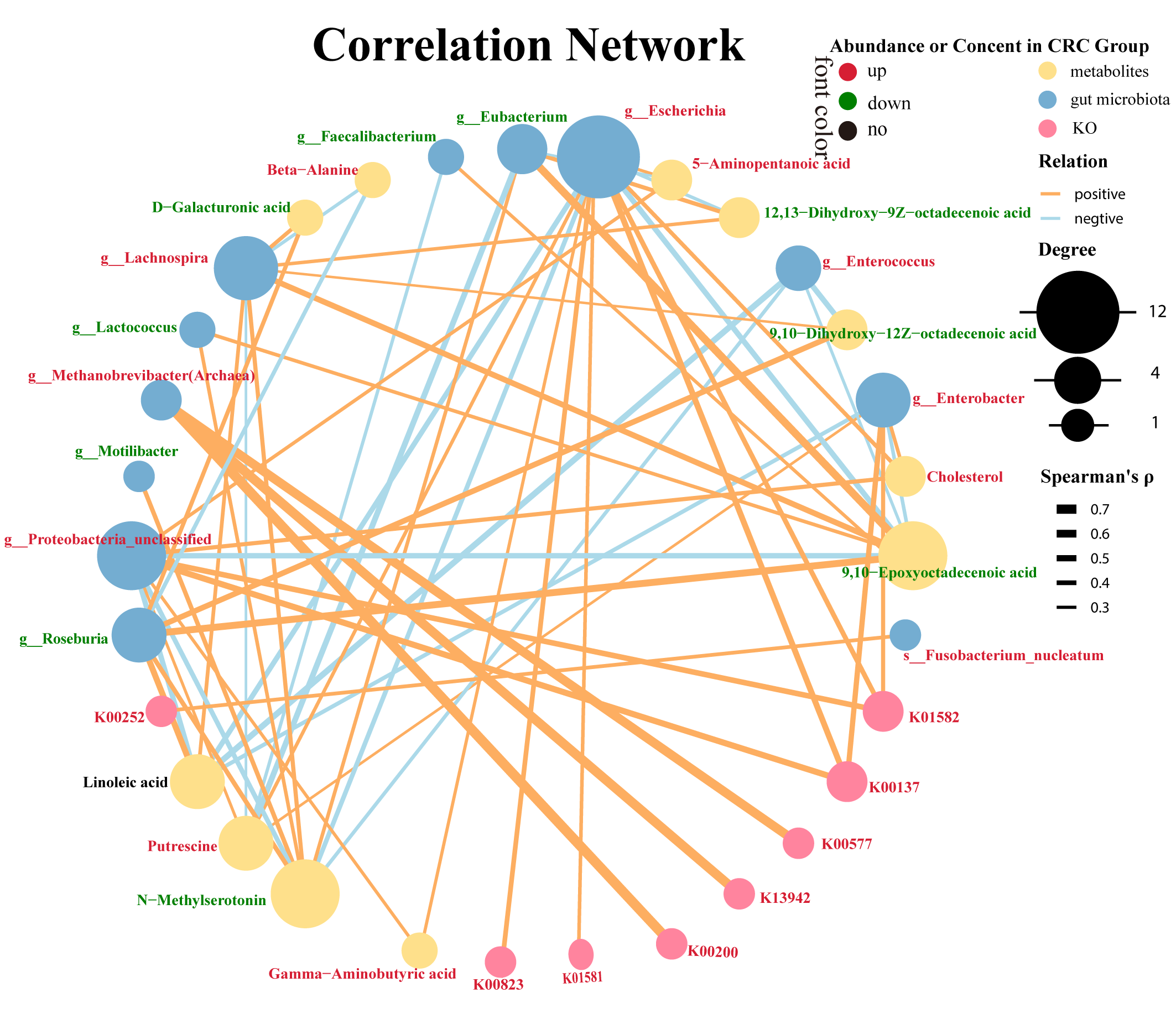


Supplementary Figure 6: Correlation network diagram of differential microbiota, differential metabolites, and KO genes.


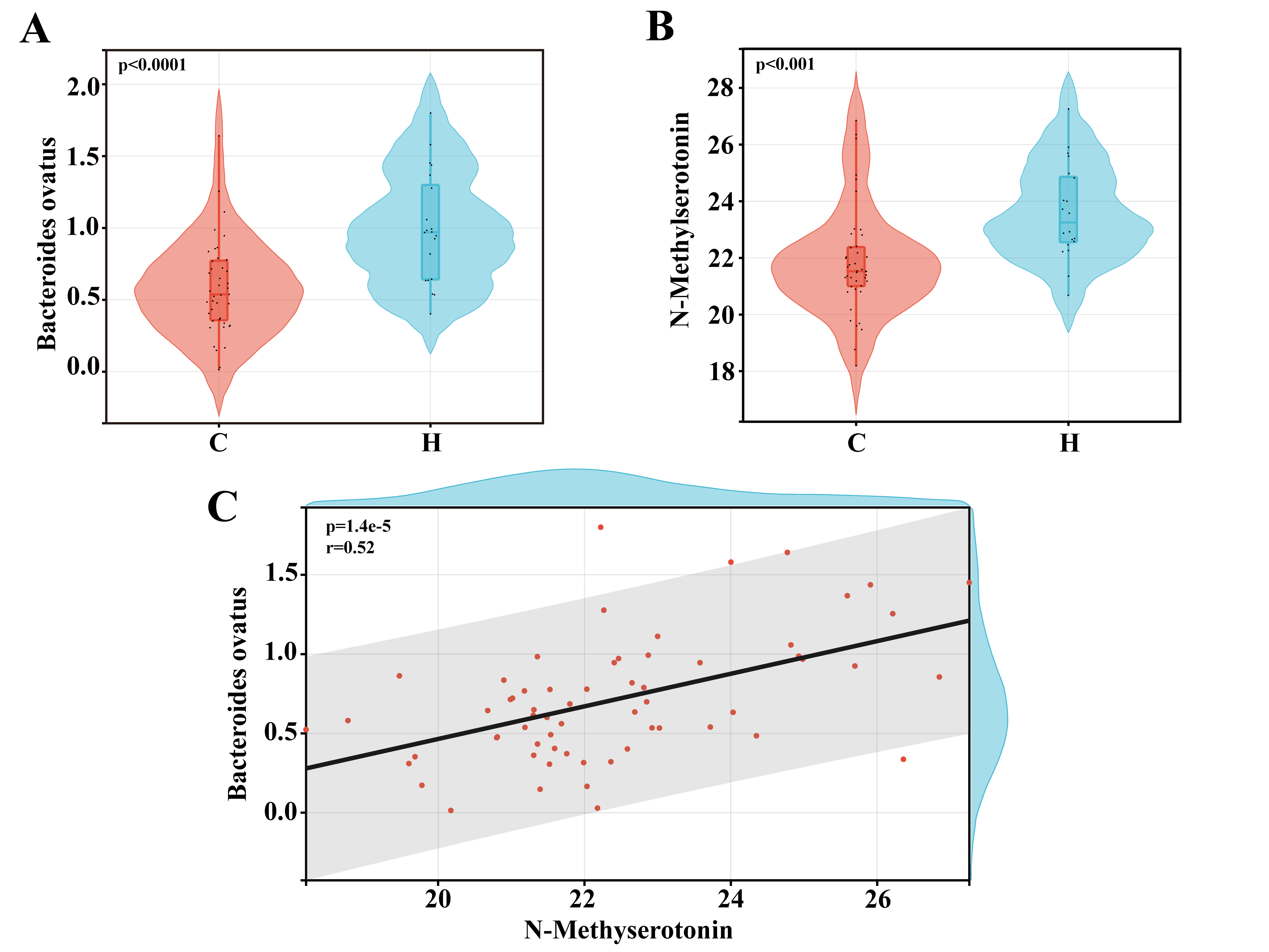


Supplementary Figure 7: (A) Differential analysis of *B.o* levels in CRC patients and healthy individuals. (B) Differential analysis of NMS levels in CRC patients and healthy individuals. (C) Correlation analysis between B.o and NMS levels.


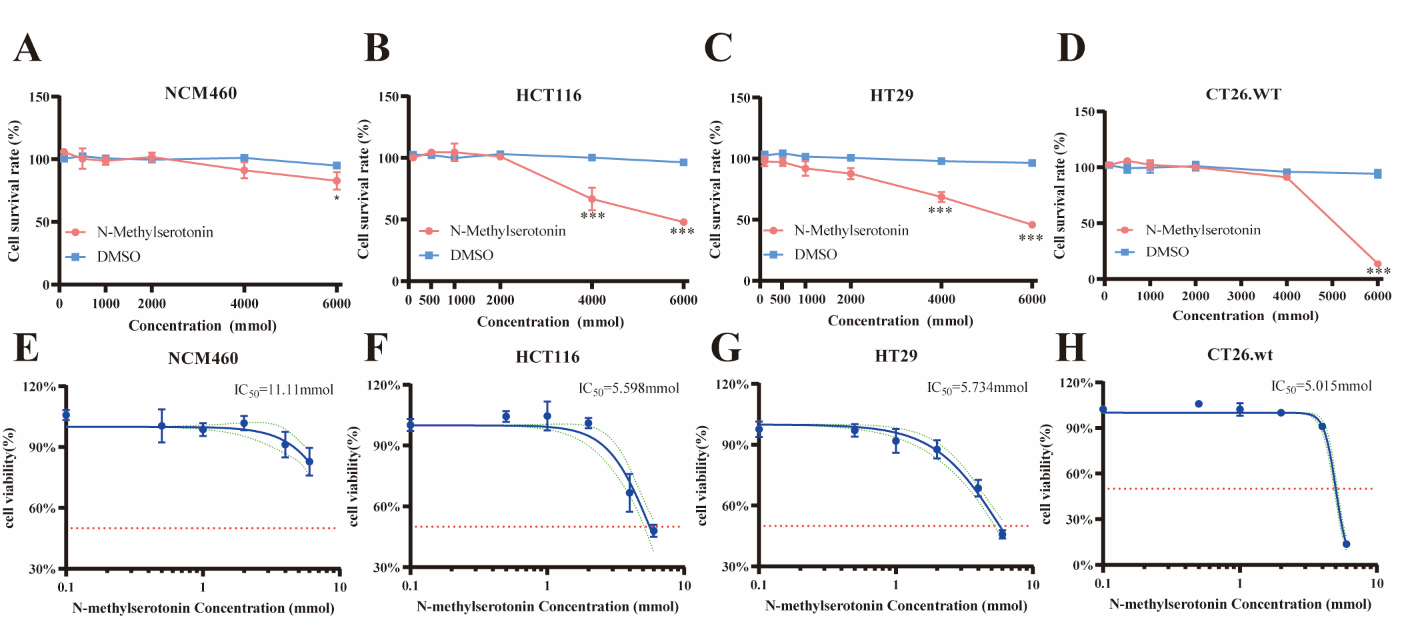


Supplementary Figure 8: CCK-8 experiments and IC_50_ values of NMS.

CCK-8 detection of the effect of NMS on the proliferation ability of NCM460 (A), HT116 (B), HT29 (C), and CT26. wt (D). NMS inhibits the IC50 values of NCM460 (E), HT116 (F), HT29 (G), and CT26. wt (H) cells.


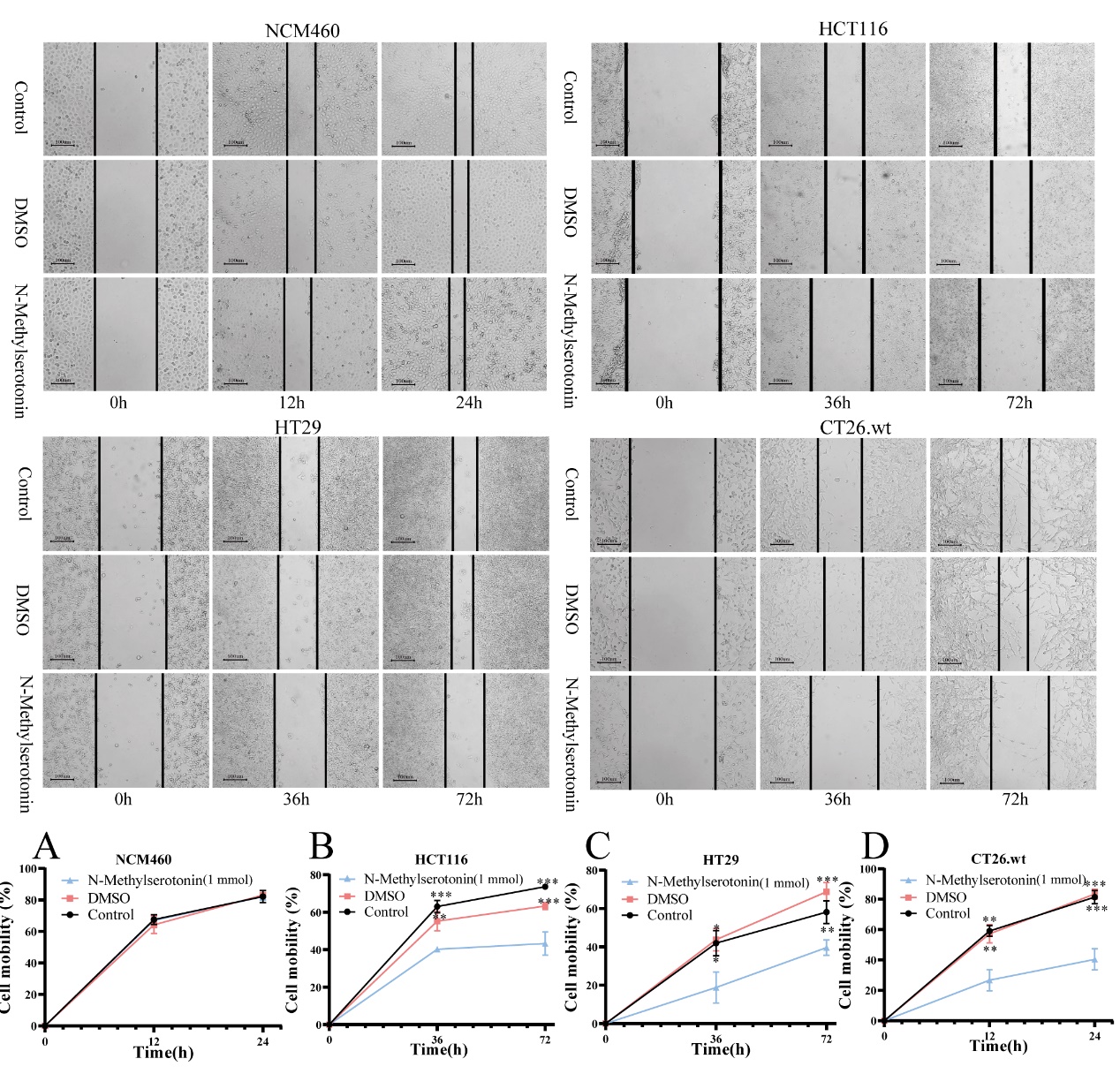


Supplementary Figure 9: Migration experiment analysis of the effect of NMS on the migration ability of NCM460 (A), HT116 (B), HT29 (C), and CT26. wt (D), (scale bar 100 µm). (** *P*≤ 0.01, *** *P*≤ 0.001).


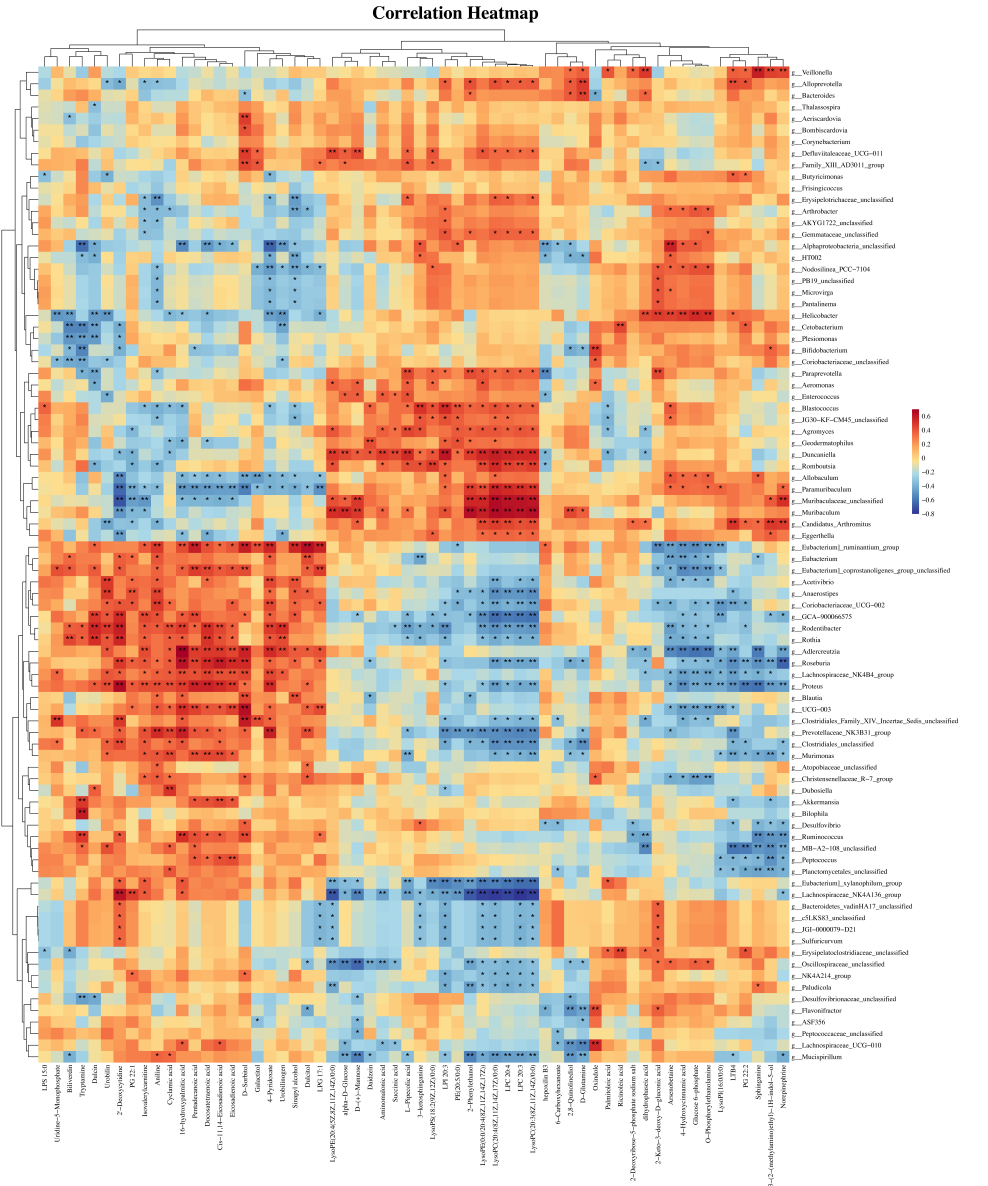


Supplementary Figure 10: Correlation heat map of differential microbiota and differential metabolites. (* *P*≤ 0.05, ** *P*≤ 0.01).
